# O‐GlcNAcylation Regulation of SNAP29‐Dependent Autophagy Activation Dictates Chemoresistance in Gastric Cancer

**DOI:** 10.1002/advs.76730

**Published:** 2026-07-23

**Authors:** Liang Tang, Shaoji Zhao, Ziling Shao, Tao Luo, Baifu Peng, Yifan Liu, Jinning Ye, Jianjun Peng, Kaiyu Sun, Jianbo Xu

**Affiliations:** ^1^ Department of Gastrointestinal Surgery The First Affiliated Hospital of Sun Yat‐Sen University Guangzhou Guangdong People's Republic of China

**Keywords:** 5‐fluorouracil, autophagy, chemoresistance, gastric cancer, O‐GlcNAcylation, oxaliplatin, SNAP29

## Abstract

Chemoresistance remains a major obstacle in gastric cancer (GC) treatment, particularly to standard fluoropyrimidine and platinum regimens. O‐GlcNAc transferase (OGT) and O‐linked β‐N‐acetylglucosamine modification (O‐GlcNAcylation) are frequently elevated in cancers, yet their specific role in GC chemotherapy remains poorly defined. Contrary to its typical oncogenic function, this study demonstrates that low OGT/O‐GlcNAcylation is associated with resistance to 5‐fluorouracil (5‐FU) or oxaliplatin (OXA) in GC. Clinically, high OGT expression correlates with better prognosis in patients receiving fluoropyrimidine/platinum chemotherapy. Functionally, OGT knockdown or O‐GlcNAcylation inhibition promotes chemoresistance both in vitro and in vivo. Mechanistically, OGT interacts with and O‐GlcNAcylates synaptosomal‐associated protein 29 (SNAP29) at Ser^2^, Ser^61^, Thr^130^, and Ser^153^. This modification inhibits STX17‐SNAP29‐VAMP8 complex assembly and autophagosome maturation. Molecular dynamics simulations suggest this involves disruption of inter‐chain hydrogen bonds and increased flexibility at SNAP29 termini. Loss of SNAP29 O‐GlcNAcylation relieves this suppression, enhances protective autophagy, and drives chemoresistance. Additionally, acute 5‐FU or OXA treatment downregulates OGT and O‐GlcNAcylation, potentially initiating a feedforward resistance loop. Importantly, elevating O‐GlcNAcylation with Thiamet‐G sensitizes GC tumors to chemotherapy in vivo. These findings reveal a context‐dependent role for the OGT/SNAP29 axis in regulating chemoresistance via autophagy and nominate OGT as a promising therapeutic target in GC.

## Graphical abstract

Model illustrating the proposed mechanism by which chemotherapy‐induced downregulation of OGT disrupts SNAP29 O‐GlcNAcylation, promoting STX17‐SNAP29‐VAMP8 SNARE complex assembly and protective autophagy, leading to chemoresistance, which in turn establishes a feedforward loop to perpetuate drug tolerance.

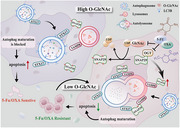

## 1 Introduction

Gastric cancer (GC) is the fifth most common cancer and the fifth leading cause of cancer‐related death globally [1]. The overall 5‐year survival rate for GC is approximately 31%, but drops sharply to only 5% upon distant metastasis [2]. Compared to surgery and targeted therapy, chemotherapy remains the primary treatment for both perioperative and advanced GC, reducing tumor recurrence and extending survival [3]. However, the development of chemoresistance severely limits its efficacy and leads to poor prognosis in GC patients [4, 5]. Doublet combinations of fluoropyrimidines and platinum are standard first‐line regimens [6], yet the specific mechanisms underlying this resistance remain unclear. Therefore, elucidating the molecular basis of fluoropyrimidine and platinum resistance in GC is crucial for identifying novel therapeutic targets and improving patient outcomes.

O‐linked β‐N‐acetylglucosamine modification (O‐GlcNAcylation/O‐GlcNAc) is a vital post‐translational modification that acts as an intracellular nutrient and stress sensor [7]. The end‐product of the hexosamine biosynthesis pathway, uridine diphosphate N‐acetylglucosamine (UDP‐GlcNAc), serves as the sole donor substrate for O‐GlcNAcylation. This dynamic modification is catalyzed by O‐GlcNAc transferase (OGT), which transfers the GlcNAc moiety to serine/threonine residues, and reversed by O‐GlcNAcase (OGA/MGEA5) [8]. Aberrant OGT expression and O‐GlcNAcylation are implicated in the development and progression of various cancers, including colorectal [9], cholangiocarcinoma [10], breast [11], and liver cancer [12]. Although OGT mRNA is reported to be elevated in GC, the expression of OGA and global O‐GlcNAcylation in GC tissues is rarely studied. Notably, emerging evidence indicates that OGT and O‐GlcNAcylation also modulate sensitivity to various anti‐cancer therapies [13, 14, 15]. For instance, RIPK1 O‐GlcNAcylation suppresses sunitinib‐induced apoptosis in renal cell carcinoma by affecting RIPK1/FADD/Caspase‐8 complex assembly and NF‐κB activation [16]. Conversely, DR4 O‐GlcNAcylation enhances TRAIL sensitivity in GC [17]. These conflicting findings underscore the urgent need to clarify the precise role and mechanisms of OGT and O‐GlcNAcylation in GC. Importantly, their function in GC resistance to 5‐fluorouracil (5‐FU) or oxaliplatin (OXA) chemotherapy remains unknown.

Autophagy involves two main stages: autophagosome initiation/formation and subsequent autophagosome maturation/degradation within autolysosomes [18]. Autophagosome‐lysosome fusion, essential for autophagic flux, requires the STX17‐SNAP29‐VAMP8 SNARE complex [19]. The role of autophagy in cancer is complex and context‐dependent. While it may suppress tumorigenesis in early stages [20], it often promotes progression and metastasis in established tumors [21]. For example, miR‐638 activates autophagy to drive proliferation and invasion in esophageal and breast cancers [22], whereas YAP1 suppresses autophagy to promote colorectal cancer growth [23]. In GC, the function of autophagy is also paradoxical. Studies show that lncRNA SNHG11 and miR‐423‐3p promote GC progression by activating autophagy [24, 25], while DAPK3 inhibits it by activating autophagy via ULK1 Ser556 phosphorylation [26]. Furthermore, the autophagy inhibitor 3‐MA reduces BDH2‐induced apoptosis [27]. Current research fails to unify the role of autophagy in GC or define the regulatory mechanisms of the STX17‐SNAP29‐VAMP8 complex in GC chemotherapy. Thus, exploring the detailed function and regulation of autophagy in GC and its response to chemotherapy is imperative.

In this study, we investigated the role and mechanism of OGT‐mediated O‐GlcNAcylation in GC resistance to 5‐FU or OXA treatment, revealing a negative regulatory interplay between 5‐FU/OXA and O‐GlcNAcylation. We demonstrate that OGT and O‐GlcNAcylation are aberrantly expressed in GC and correlate with the prognosis of patients receiving fluoropyrimidine/platinum‐based chemotherapy. Downregulation of OGT and O‐GlcNAcylation promoted resistance to 5‐FU or OXA both in vitro and in vivo, whereas acute 5‐FU/OXA treatment suppressed O‐GlcNAcylation. Mechanistically, OGT knockdown reduced O‐GlcNAcylation of SNAP29 at Ser^2^, Ser^61^, Thr^130^, and Ser^153^, as validated by single‐site and quadruple mutagenesis. This hypo‐O‐GlcNAcylation facilitated the assembly of the STX17‐SNAP29‐VAMP8 complex, enhanced autophagic flux, and thereby drove chemoresistance. Molecular dynamics simulations further suggested that this effect involves disruption of inter‐chain hydrogen bonds and increased conformational flexibility at the SNAP29 termini. Rescue experiments confirmed that these four sites are functionally required for SNAP29‐mediated autophagy and chemoresistance. Acute 5‐FU/OXA treatment also downregulated UDP‐GlcNAc and OGT levels, suppressing global O‐GlcNAcylation and potentially exacerbating a vicious cycle of resistance. Our findings elucidate the functional role and mechanism of OGT‐mediated O‐GlcNAcylation in GC 5‐FU/OXA chemoresistance, highlighting its potential as a promising therapeutic target.

## 2 Results

### 2.1 OGT Downregulation Is Associated With Chemoresistance and Poor Survival in GC

**FIGURE 1:**
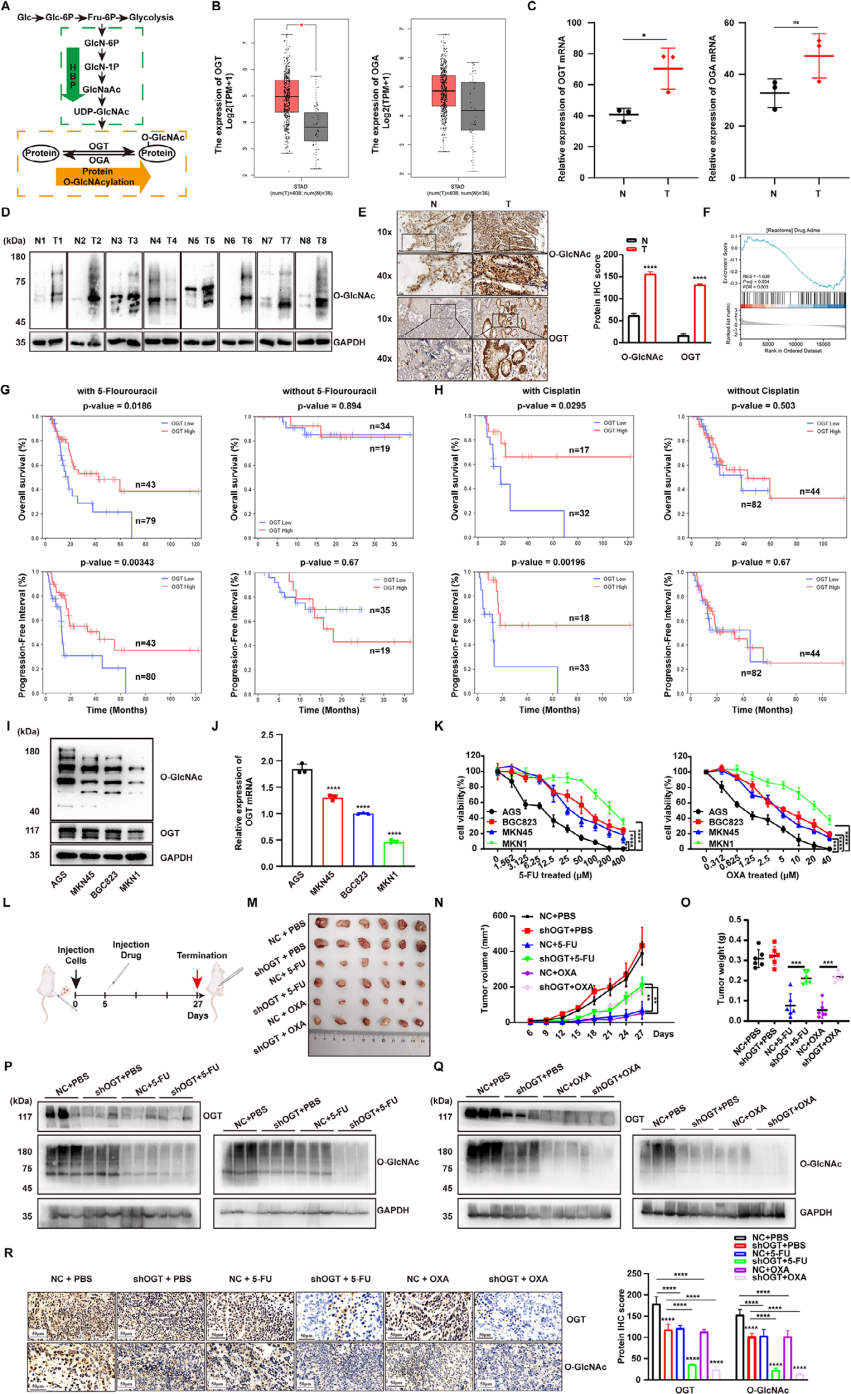
OGT Downregulation is Associated with Chemoresistance and Poor Survival in GC. (A) Schematic diagram summarizing the glucose metabolic pathway, HBP, and protein O‐GlcNAcylation. (B) Expression of OGT and OGA in GC and adjacent normal tissues based on the TCGA database. (C) Expression of OGT and OGA in GC and adjacent normal tissues based on our three paired RNA sequencing data. (D) Expression levels of O‐GlcNAcylation in eight pairs of GC and adjacent tissues by WB. (E) Representative IHC images of O‐GlcNAcylation and OGT in GC and adjacent tissues. Scale bars: 25–100 µm. (F) GSEA reveals a negative correlation between high OGT expression in GC from the TCGA database and the Drug ADME (Absorption, Distribution, Metabolism, and Excretion) pathway. (G, H) Kaplan–Meier analysis of OS and PFI in GC patients from the TCGA database, stratified by OGT expression (high vs low). Patients treated with 5‐FU or cisplatin (left) and patients who did not receive this specific regimen (right) are shown separately. (I) Protein expression levels of OGT and O‐GlcNAcylation in various GC cells. (J) Expression of OGT mRNA in various GC cells. (K) CCK‐8 assay comparing the cell viability between various GC cells after being treated with 5‐FU (left) or OXA (right). (L) Schematic illustration of the MKN45‐line‐derived subcutaneous xenograft mouse model. (M) 1 × 10^6^ NC and shOGT MKN45 cells were subcutaneously injected into nude mice (n = 6). Mice were treated for 27 days, and tumors were collected for imaging. (N) Tumor volume was recorded every 3 days. (O) Tumor weight was measured after 27 days. (P, Q) WB analysis of O‐GlcNAcylation levels in tumor tissues from the indicated treatment groups. Samples from mice treated with 5‐FU (P) or OXA (R). (R) IHC analysis of OGT and O‐GlcNAcylation in tumor sections using indicated antibodies (Scale bars: 50 µm). Data are expressed as mean ± SD of biological replicate experiments. *p < 0.05, **p < 0.01, ***p < 0.001, ****p < 0.0001.

The hexosamine biosynthesis pathway (HBP) is closely linked to cancer, with O‐GlcNAc transferase (OGT) and O‐GlcNAcase (OGA) mediating protein O‐linked β‐N‐acetylglucosamine modification (O‐GlcNAcylation) and de‐O‐GlcNAcylation, respectively (Figure 1A). To investigate the role of O‐GlcNAcylation in GC progression, we analyzed OGT and OGA expression levels in GC and adjacent normal tissues using the Cancer Genome Atlas (TCGA) database. The results showed that OGT expression was upregulated in GC tissues, while OGA showed no significant difference (Figure 1B). To validate this finding, we analyzed transcriptomic sequencing data from three paired GC and adjacent non‐tumor tissues. Consistent with the TCGA results, OGT mRNA expression was significantly upregulated in GC tissues compared to non‐tumor tissues, whereas OGA expression showed no statistical difference (Figure 1C). To determine whether elevated OGT expression in GC mediates increased O‐GlcNAcylation, we also examined OGT mRNA and global O‐GlcNAcylation levels in eight paired GC tissue samples and their adjacent non‐tumor counterparts. Consistent with the OGT mRNA results (Figure S1A), WB confirmed that O‐GlcNAcylation was also upregulated in GC tissues compared to non‐tumor tissues (Figure 1D). Furthermore, we performed immunohistochemical (IHC) staining on pathological sections from an additional 80 paired gastric cancer and adjacent normal tissue samples. The IHC results consistently demonstrated elevated expression of both OGT and O‐GlcNAcylation in gastric cancer tissues (Figure 1E). Collectively, these findings indicate aberrant expression of OGT and its mediated O‐GlcNAcylation in GC.

We analyzed TCGA data and the Kaplan‐Meier plotter to assess the prognostic value of OGT and OGA in GC. However, survival analysis revealed that neither OGT nor OGA expression levels were significantly associated with patient prognosis (Figure S1B,C). Intriguingly, a previous study found that DR4‐Ser424 O‐GlcNAcylation promoted sensitivity to TRAIL therapy in GC [17]. We further performed Gene Set Enrichment Analysis (GSEA) on TCGA‐STAD samples, which showed that high OGT expression was negatively correlated with chemoresistance‐related pathways [28, 29, 30, 31] such as drug metabolism, drug transport, and epithelial mesenchymal transition (EMT) (Figure 1F; Figure S1D–G), suggesting OGT might promote drug sensitivity. To evaluate whether OGT affects the survival of GC patients receiving drug therapy, we used GEPIA3 (https://gepia3.bioinfoliu.com) to analyze the prognostic value of OGT and OGA expression in GC patients from the TCGA database who received chemotherapy. The results suggested that high OGT expression was associated with better overall survival (OS) and progression‐free interval (PFI) in GC patients treated specifically with 5‐fluorouracil (5‐FU) or cisplatin, but not in patients who did not receive this regimen (Figure 1G,H). In clinical practice, the newer platinum agent oxaliplatin (OXA) is more widely used in GC treatment due to its superior tolerability [32, 33]. Considering the possibility that the small sample size of OXA‐treated GC patients in the TCGA database might obscure statistical significance, we next investigated the correlation between OGT and sensitivity to 5‐FU or OXA chemotherapy in GC cell lines. We first determined the abundance of OGT expression in GC cell lines. WB and qPCR results showed the highest expression in AGS, followed by MKN45 and BGC823, and the lowest in MKN1 (Figure 1I,J). CCK‐8 assays revealed that dose‐response curves were significantly right‐shifted in cells with low OGT expression, indicating that cell lines with higher OGT expression were more sensitive to chemotherapy (Figure 1K). To validate this finding in vivo, we established a subcutaneous xenograft model in nude mice using MKN45 GC cells with stable OGT knockdown obtained via fluorescence‐activated cell sorting (Figure 1L). Mice were divided into six groups: NC, shOGT, NC + 5‐FU, shOGT + 5‐FU, NC + OXA, and shOGT + OXA. Subsequently, mice received daily intraperitoneal injections of 5‐FU or OXA and were sacrificed one month later to harvest the subcutaneous tumors. The results demonstrated that OGT knockdown increased tumor volume and weight following treatment with either 5‐FU or OXA (Figure 1M–O). To elucidate the relationship between O‐GlcNAcylation levels and chemosensitivity, we assessed global O‐GlcNAcylation in the tumor tissues. Both WB and IHC showed lower O‐GlcNAcylation modification in the OGT‐knockdown groups (Figure 1P–R). Furthermore, compared to the non‐chemotherapy groups, the 5‐FU or OXA treatment groups also exhibited reduced O‐GlcNAcylation (Figure 1P–R). Overall, these findings indicate that OGT knockdown promotes GC resistance to 5‐FU or OXA.

### 2.2 5‐FU or OXA Reduce OGT and O‐GlcNAcylation in GC Cells

**FIGURE 2:**
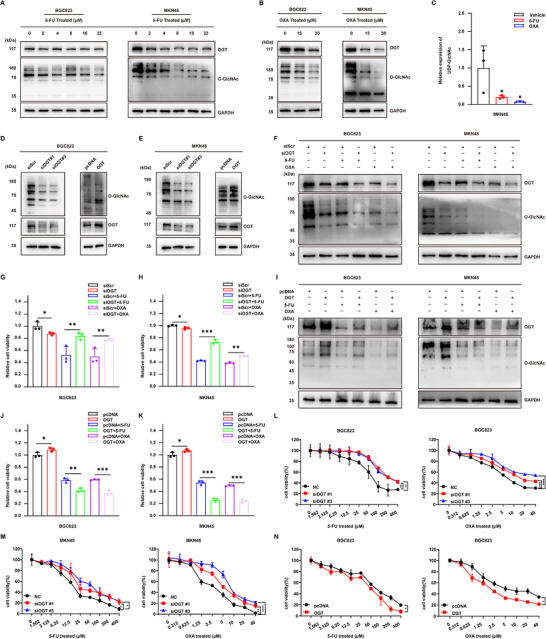
5‐FU or OXA Reduces OGT and O‐GlcNAcylation in GC Cells. (A, B) WB analysis of OGT and O‐GlcNAcylation in BGC823 and MKN45 cells following treatment with increasing concentrations of 5‐FU (A) or OXA (B). (C) Fold change of UDP‐GlcNAc following treatment with increasing concentrations of 5‐FU or OXA. (D, E) The efficiency of OGT knockdown and overexpression was verified in BGC823 (D) and MKN45 (E) cells by Western blot analysis. (F–H) BGC823 and MKN45 cells were transfected with scrambled siRNA (siScr) or OGT siRNA (siOGT) for 48 h, then treated with 5‐FU or OXA. Protein expression was evaluated by WB (F), and cell viability was measured using the CCK‐8 assay (G, H). (I–K) BGC823 and MKN45 cells were transfected with control (pcDNA) or OGT‐overexpressing vectors for 48 h, then treated with 5‐FU or OXA. Protein expression was evaluated by WB (I), and cell viability was measured using the CCK‐8 assay (J, K). (L) CCK‐8 assays comparing the viability of BGC823 cells transfected with NC or siOGT, following treatment with 5‐FU (left) or OXA (right). (M) CCK‐8 assays comparing the viability of MKN45 cells transfected with NC or siOGT, following treatment with 5‐FU (left) or OXA (right). (N) CCK‐8 assays comparing the viability of BGC823 cells transfected with either an empty vector (pcDNA) or an OGT‐overexpression plasmid, following treatment with 5‐FU (left) or OXA (right). Data are expressed as mean ± SD of biological replicate experiments. *p < 0.05, **p < 0.01, ***p < 0.001, ****p < 0.0001.

Given the observed effects of 5‐FU or OXA on OGT and O‐GlcNAcylation expression in vivo, we validated these findings in vitro. The MKN45 and BGC823 cell lines, which exhibit moderate endogenous OGT/O‐GlcNAcylation levels and drug sensitivity, were selected for subsequent experiments. Treatment of MKN45 and BGC823 cells with increasing concentrations of 5‐FU or OXA alone for 24 h resulted in a dose‐dependent decrease in both OGT and global O‐GlcNAcylation levels, as shown by WB (Figure 2A,B). qPCR analysis confirmed that OGT mRNA was significantly suppressed (Figure S2A,B). Furthermore, untargeted metabolomics revealed that the metabolic substrate required for O‐GlcNAcylation, UDP‐GlcNAc, was also downregulated following chemotherapy treatment (Figure 2C). We next investigated the impact of further OGT knockdown on the proliferative viability of GC cells post‐chemotherapy. The efficiency of OGT knockdown and overexpression in the two GC cell lines was confirmed at both mRNA and protein levels (Figure S2C–F; Figure 2D,E). After 48 h of chemotherapy treatment, WB and CCK‐8 assays showed that OGT knockdown further reduced global O‐GlcNAcylation and enhanced the proliferative viability of GC cells under chemotherapy (Figure 2F,G). Conversely, OGT overexpression attenuated the proliferative viability of GC cells upon chemotherapy exposure (Figure 2I,J). Dose‐response curves indicated a rightward shift following OGT knockdown and a leftward shift following OGT overexpression (Figure 2L–N). To validate the specificity of OGT knockdown‐induced chemoresistance, we re‐expressed OGT in shOGT cells. The results showed that chemosensitivity was restored in the shOGT+OGT group, as evidenced by a leftward shift of the dose‐response curves (Figure S2G,H). Therefore, these results demonstrate that chemotherapy reduces OGT expression and O‐GlcNAcylation in GC cells in vitro, and that further OGT knockdown promotes GC cell survival under chemotherapy.

### 2.3 Inhibition of O‐GlcNAcylation Reduces 5‐FU or OXA Induced Apoptosis and Promotes Chemoresistance in GC Cells

**FIGURE 3:**
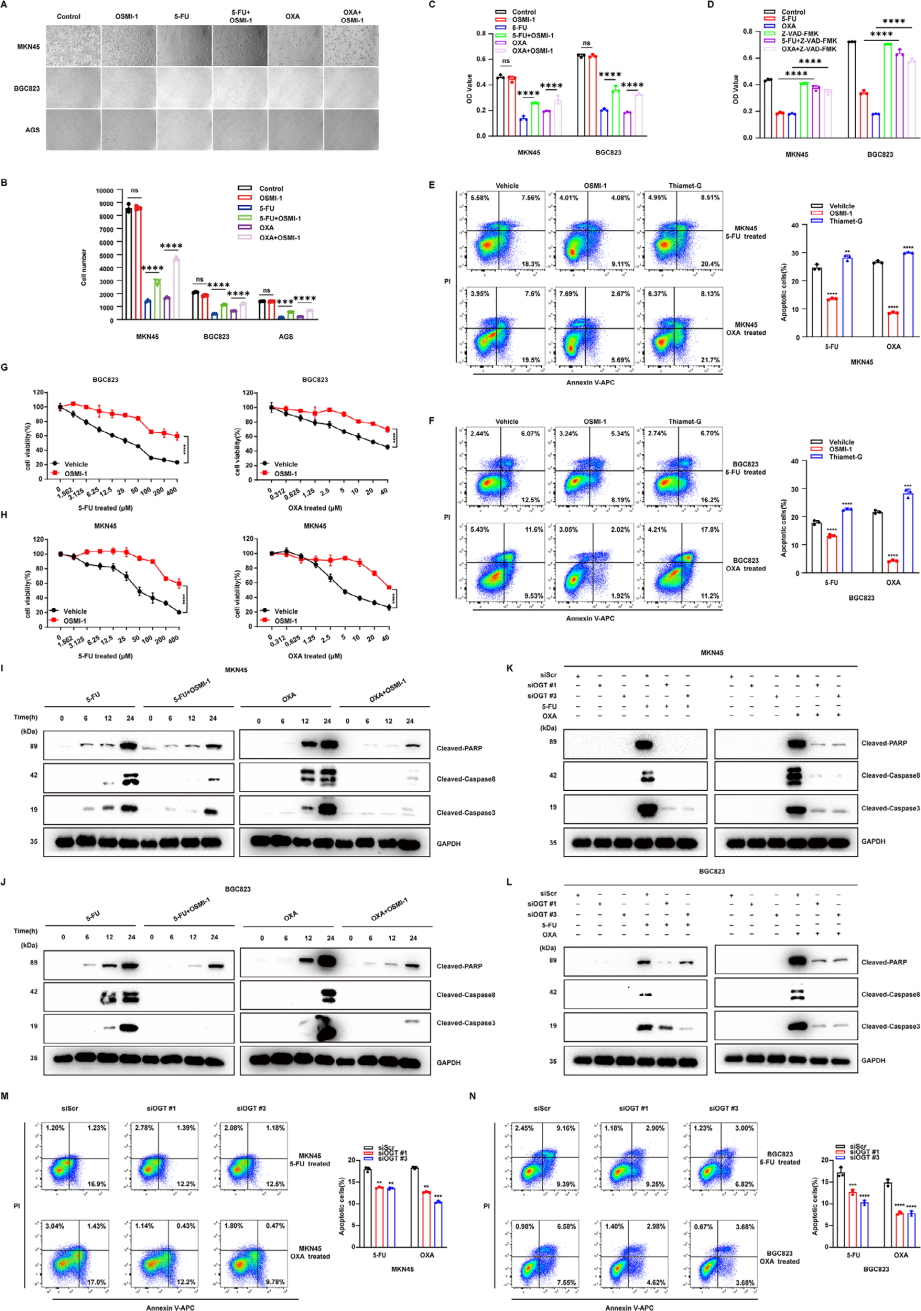
Inhibition of O‐GlcNAcylation Reduces 5‐FU or OXA‐Induced Apoptosis and Promotes Chemoresistance in GC Cells. (A) Representative bright‐field images of MKN45, BGC823, and AGS cells exposed to 5‐FU/OXA with or without OSMI‐1. (B) Quantitative results corresponding to Figure A. (C) CCK8 assays for MKN45 and BGC823 cells exposed to 5‐FU/OXA with or without OSMI‐1. (D) CCK8 assays for MKN45 and BGC823 cells exposed to 5‐FU/OXA with or without apoptosis inhibitor. (E, F) Analysis of apoptosis by flow cytometry. MKN45 (E) and BGC823 (F) cells were treated with vehicle, OSMI‐1, or Thiamet‐G following exposure to 5‐FU or OXA. Apoptosis was assessed using APC‐Annexin V and PI staining. Representative flow cytometry plots (left) and quantitative analysis (right) of apoptotic cells under the indicated conditions. (G, H) CCK‐8 assays comparing the viability of BGC823 (G) and MKN45 (H) cells treated with vehicle or OSMI‐1, following exposure to increasing concentrations of 5‐FU (left) or OXA (right). (I, J) MKN45 (I) and BGC823 (J) cells were exposed to 5‐FU/OXA with or without OSMI‐1 for the indicated time. Apoptosis markers, including cleaved caspase‐3, cleaved caspase‐8, and cleaved PARP, levels were examined by WB. (K, L) MKN45 (K) and BGC823 (L) cells transfected with NC or siOGT were exposed to 5‐FU or OXA for the indicated time. Apoptosis markers, including cleaved caspase‐3, cleaved caspase‐8 and cleaved PARP, levels were examined by WB. (M,N) Analysis of apoptosis by flow cytometry. MKN45 (M) and BGC823 (N) cells were transfected with NC or siOGT following exposure to 5‐FU or OXA. Apoptosis was assessed using APC‐Annexin V and PI staining. Representative flow cytometry plots (left) and quantitative analysis (right) of apoptotic cells under the indicated conditions. Data are expressed as mean ± SD of biological replicate experiments. **p < 0.01, ***p < 0.001, ****p < 0.0001.

To investigate the critical role of O‐GlcNAcylation in 5‐FU or OXA sensitivity, we employed the small‐molecule inhibitor OSMI‐1 [34] and activator Thiamet‐G [35], confirming their efficacy in modulating global O‐GlcNAcylation (Figure S3A,B). The on‐target specificity of Thiamet‐G was supported by genetic validation showing that siOGA similarly elevated O‐GlcNAcylation levels (Figure S3C), and by dose‐response data demonstrating concentration‐dependent O‐GlcNAcylation elevation without cytotoxicity up to 10 µM (Figure S3D,E). Inhibition of O‐GlcNAcylation significantly reduced cell death induced by 5‐FU or OXA (Figure 3A–C). Furthermore, the apoptosis inhibitor Z‐VAD‐FMK markedly diminished 5‐FU/OXA‐induced programmed cell death (Figure 3D), indicating that this cell death occurred primarily via apoptosis. Flow cytometry analysis and CCK‐8 assays showed that O‐GlcNAcylation inhibition significantly reduced apoptosis (Figure 3E,F; Figure S3F) and caused a marked rightward shift in the dose‐response curves of GC cells to 5‐FU or OXA (Figure 3G,H), indicating decreased drug sensitivity. Conversely, the activator Thiamet‐G produced the opposite effects (Figure 3E,F; Figure S3G). We also examined the expression of apoptosis markers following 5‐FU/OXA treatment. WB results demonstrated that O‐GlcNAcylation inhibition significantly downregulated cleaved Caspase‐3, cleaved Caspase‐8, and cleaved PARP (Figure 3I,J; Figure S3H,I). These results indicate that inhibition of O‐GlcNAcylation attenuates 5‐FU or OXA induced apoptosis in GC cells. Consistently, apoptosis‐related protein expression (Figure 3K,L) and flow cytometry analysis (Figure 3M,N; Figure S3J) in OGT‐knockdown cells aligned with the effects of pharmacological O‐GlcNAcylation inhibition.

### 2.4 Autophagy Activation Is Crucial for OGT‐Knockdown‐Mediated Chemoresistance in GC

**FIGURE 4:**
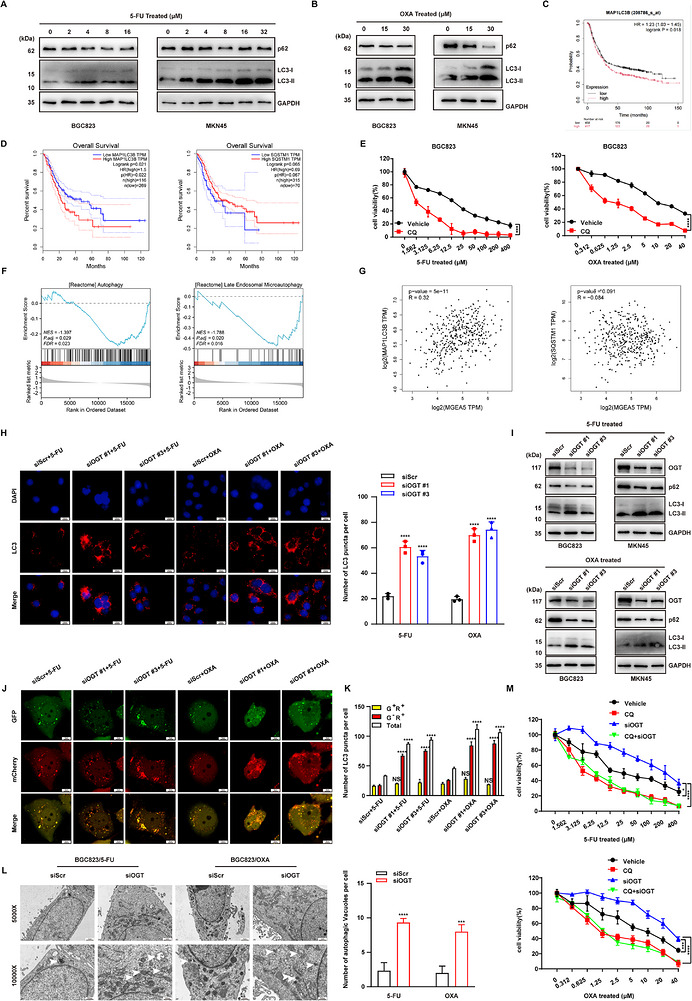
Autophagy Activation is Crucial for OGT‐Knockdown‐Mediated Chemoresistance in GC. (A, B) BGC823 and MKN45 cells were treated with the indicated concentrations of 5‐FU (A) or OXA (B) for 24 h. Protein levels of LC3‐I, LC3‐II, and p62 were examined by WB. (C) Kaplan–Meier curves of OS of patients with GC based on the LC3 expression levels. (D) Kaplan–Meier analysis of OS in GC patients from the TCGA database, stratified by LC3 and p62 expression (high vs low). (E) CCK‐8 assays comparing the viability of BGC823 cells treated with CQ, following exposure to increasing concentrations of 5‐FU (left) or OXA (right). (F) GSEA reveals a negative correlation between high OGT expression in GC from the TCGA database and the autophagy pathway. (G) Correlation analysis between OGA expression and LC3, p62 expression in GC from the TCGA database. (H) Representative IF images (left) and quantification (right) of LC3 puncta in BGC823 cells transfected with NC or siOGT following treatment with 5‐FU or OXA. Scale bars = 10 µm, Images were acquired using a 40× objective. (I) WB analysis of LC3‐I, LC3‐II, and p62 in NC or siOGT‐transfected cells following treatment with 5‐FU (upper) or OXA (lower). (J, K) Representative fluorescence images of RFP‐GFP‐LC3‐transfected NC and siOGT BGC823 cells treated with 5‐FU or OXA (scale: 5 µm, Images were acquired using a 60× objective) (J), and quantitative analysis of GFP^+^RFP^+^, GFP^−^RFP^+^ puncta (K). (L) TEM of NC and siOGT BGC823 cells treated with 5‐FU or OXA. White arrows indicate the autophagic Vacuoles (scale bars: 500 nm–1 µm, magnification 5000× – 10000×) (left). Quantification of autophagosome numbers (rigth). (M) CCK‐8 assays comparing the viability of NC and siOGT‐transfected BGC823 cells co‐treated with CQ and increasing concentrations of 5‐FU (upper) or OXA (lower). Data are expressed as mean ± SD of biological replicate experiments. *p < 0.05, ***p < 0.001, ****p < 0.0001.

Autophagy is widely implicated in cancer chemoresistance [18, 36]. To investigate the relationship between chemotherapy and autophagy, GC cells were treated with increasing concentrations of 5‐FU or OXA for 24 h. Autophagy levels were evaluated by examining the expression of MAP1LC3B (LC3: detecting both LC3‐I and LC3‐II forms), and SQSTM1 (p62). WB results showed that chemotherapy activated autophagy in a dose‐dependent manner (Figure 4A,B). Analysis of Kaplan‐Meier plotter and TCGA‐STAD data revealed that high LC3 expression correlated with poorer OS, whereas high p62 expression was associated with better OS (Figure 4C,D). Treating GC cells with the autophagy inhibitor chloroquine (CQ) in combination with chemotherapy for 48 h resulted in a leftward shift of the dose‐response curves in the CCK‐8 assay, suggesting that chemotherapy‐induced autophagy may promote GC cell survival (Figure 4E). Previous studies indicate that OGT knockdown or O‐GlcNAcylation blockade can activate autophagy. Our prior findings also show that chemotherapy suppresses OGT and O‐GlcNAcylation. Based on this, we hypothesized that autophagy activation participates in the chemoresistance mediated by reduced O‐GlcNAcylation in GC cells. Re‐analysis of our GSEA results from TCGA‐STAD samples revealed that high OGT expression was significantly negatively correlated not only with autophagy (Figure 4F) and related pathways, but also with common chemoresistance‐associated pathways, particularly base excision repair and nucleotide excision repair (Figure S4A), which mediate fluoropyrimidine and platinum resistance [37, 38]. Furthermore, OGA expression showed a significant positive correlation with key positive regulators of autophagy (LC3, ULK1, BECN1, LAMP2) (Figure 4G; Figure S4B) and a negative correlation with p62 (Figure 4G), a protein negatively associated with autophagic flux. These results suggest that O‐GlcNAcylation inhibition may be linked to enhanced autophagy and chemoresistance. IF staining confirmed that OGT knockdown increased the number and intensity of LC3 puncta in GC cells (Figure S4C). WB analysis showed that OGT knockdown elevated LC3‐II/LC3‐I ratio while reducing p62 expression (Figure S4D), indicating enhanced basal autophagy. To examine how OGT knockdown regulates autophagy under chemotherapy, GC cells were treated with 5‐FU or OXA. IF staining revealed a more significant increase in LC3 puncta number and intensity in OGT‐knockdown cells compared to controls following chemotherapy (Figure 4H; Figure S4C)). Similarly, WB demonstrated a greater increase in LC3‐II/LC3‐I ratio and decrease in p62 in OGT‐knockdown cells (Figure 4I), an effect that was amplified by 5‐FU or OXA treatment (Figure S4D). To directly assess autophagic flux, we employed the RFP‐GFP‐LC3 reporter system. Since GFP fluorescence is quenched in acidic lysosomes, autophagosomes appear as GFP^+^RFP^+^ puncta, whereas autolysosomes appear as GFP^−^RFP^+^ puncta [39]. Confocal microscopy analysis showed that in GC cells treated with 5‐FU or OXA, OGT knockdown significantly increased the number of GFP^−^RFP^+^ puncta (autolysosomes) and the total number of LC3 puncta, while the number of GFP^+^RFP^+^ puncta (autophagosomes) remained relatively unchanged (Figure 4J,K). This trend was minimal in untreated cells (Figure S4E,F). CQ treatment further confirmed that OGT knockdown promotes autophagic flux, as evidenced by a marked increase in GFP^+^RFP^+^ puncta and total LC3 puncta upon CQ treatment (Figure S4G,H). Transmission electron microscopy (TEM) further confirmed that OGT knockdown markedly increased the number of autophagic vesicles in GC cells after 5‐FU or OXA treatment (Figure 4L). To determine whether autophagy activation is required for OGT‐knockdown‐dependent chemoresistance, we used CQ to inhibit autophagy. CCK‐8 assays showed that CQ treatment caused a leftward shift in the dose‐response curves and completely abolished the pro‐resistance effect conferred by OGT knockdown (Figure 4M). Flow cytometry further showed that CQ reversed the anti‐apoptotic effect induced by OGT knockdown (Figure S4I). In summary, our results demonstrate that OGT knockdown significantly enhances autophagy during chemotherapy, and that this autophagic activation mediates the regulatory role of OGT knockdown in GC chemoresistance.

### 2.5 OGT Knockdown Increases Autophagy and Enhances Resistance to 5‐FU or OXA by Inhibiting SNAP29 O‐GlcNAcylation at Ser^2^, Ser^61^, Thr^130^, and Ser^153^

**FIGURE 5:**
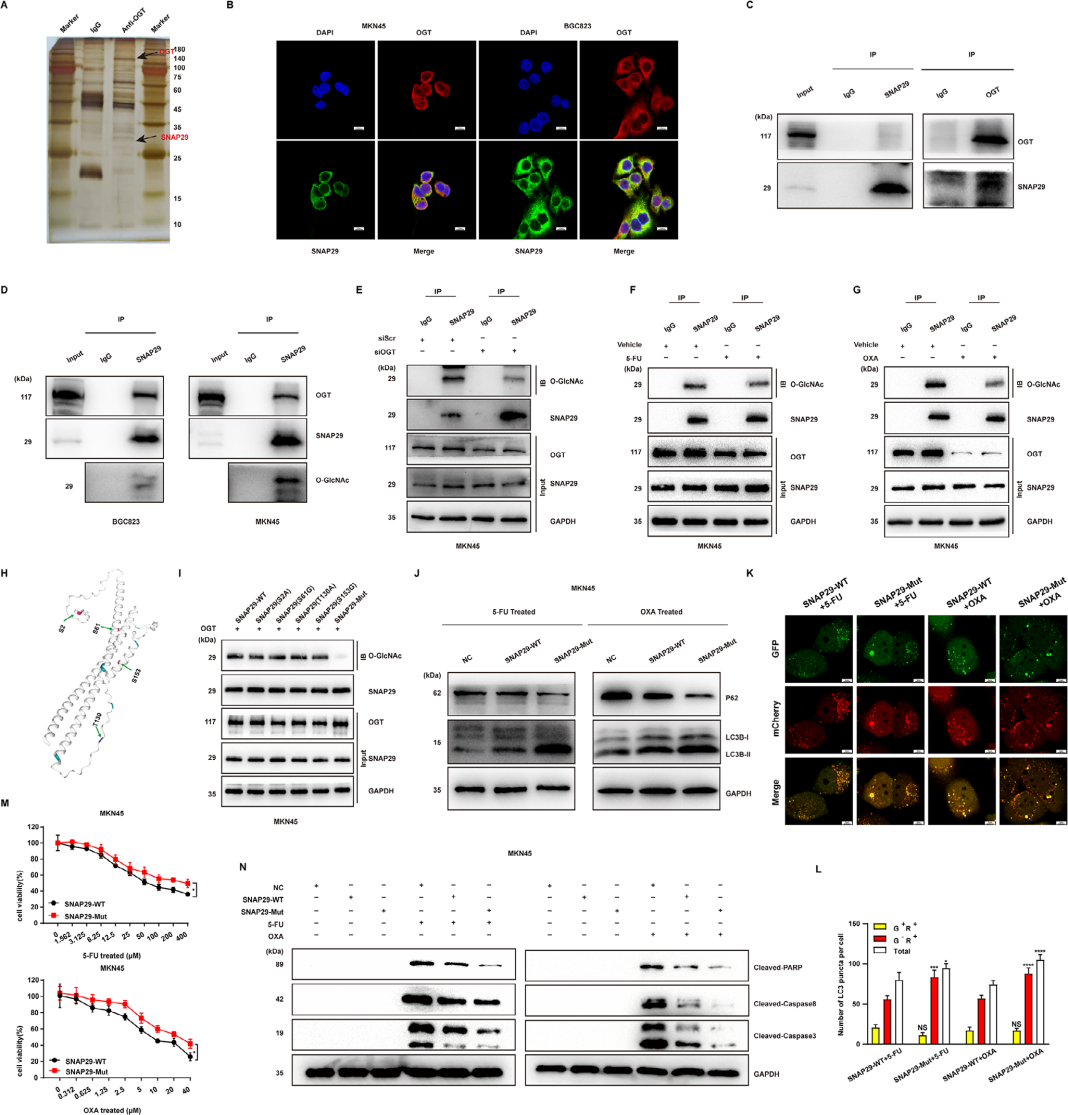
OGT Knockdown Increases Autophagy and Enhances Resistance to 5‐FU or OXA by Inhibiting SNAP29 O‐GlcNAcylation at Ser^2^, Ser^61^, Thr^130^, and Ser^153^. (A) Co‐IP assay using an anti‐OGT antibody, followed by protein separation via SDS‐PAGE and silver staining. (B) The cytoplasmic co‐localization of OGT and SNAP29 in MKN45 and BGC823 cells was confirmed by IF (scale bars: 10 µm). (C, D) Endogenous Co‐IP showing OGT‐SNAP29 interaction (D) and SNAP29 O‐GlcNAcylation (D) in GC cells. (E) IP assay comparing SNAP29 O‐GlcNAcylation in NC‐ and siOGT‐transfected MKN45 cells. (F, G) IP assay comparing SNAP29 O‐GlcNAcylation in MKN45 cells treated with or without 5‐FU (F) or OXA (G). (H) Potential O‐GlcNAcylation sites on SNAP29 predicted using the YinOYang database. (I) Comparing SNAP29 O‐GlcNAcylation in MKN45 cells co‐transfected with OGT‐ovIP assay erexpressing vector and SNAP29‐WT, single‐site mutants (S2A, S61G, T130A, S153G), or quadruple mutant (SNAP29‐Mut). (J) WB analysis of LC3‐I, LC3‐II, and p62 in MKN45 cells transfected with NC, SNAP29‐WT, or SNAP29‐Mut and subsequently treated with 5‐FU or OXA. (K, L) Representative fluorescence images of RFP‐GFP‐LC3‐transfected SNAP29‐WT and SNAP29‐Mut GC cells treated with 5‐FU or OXA (scale: 5 µm, Images were acquired using a 60× objective) (K), and quantitative analysis of GFP^+^RFP^+^, GFP^−^RFP^+^ puncta (L). (M) CCK‐8 assays comparing the viability of MKN45 cells transfected with SNAP29‐WT or SNAP29‐Mut, following treatment with 5‐FU or OXA. (N) MKN45 cells transfected with SNAP29‐WT or SNAP29‐Mut were exposed to 5‐FU or OXA for indicated time. Apoptosis markers, including cleaved caspase‐3, cleaved caspase‐8, and cleaved PARP, levels were examined by WB. Data are expressed as mean ± SD of biological replicate experiments. *p < 0.05, ***p < 0.001, ****p < 0.0001.

To elucidate the specific mechanism by which OGT knockdown promotes autophagy activation, we performed co‐immunoprecipitation (Co‐IP) assays in MKN45 GC cells using anti‐OGT or IgG antibodies. Silver staining revealed a specific band at approximately 30 kDa in the anti‐OGT group, and subsequent mass spectrometry (MS) identified Synaptosomal‐Associated Protein 29 (SNAP29) as one of the most abundant proteins (Figure 5A). IF showed that OGT was distributed in both the cytoplasm and nucleus, and SNAP29 exhibited clear co‐localization with OGT in the cytoplasm (Figure 5B). Consistently, endogenous Co‐IP assays in two GC cell lines confirmed a specific interaction between OGT and SNAP29, and SNAP29 was found to be O‐GlcNAcylated (Figure 5C,D). To determine whether OGT promotes SNAP29 O‐GlcNAcylation, we performed IP with anti‐SNAP29 antibody following OGT knockdown. The results showed that OGT knockdown downregulated SNAP29 O‐GlcNAcylation without affecting SNAP29 protein expression (Figure 5E; Figure S5A). To quantitatively assess the effect of chemotherapy on SNAP29 O‐GlcNAcylation, we performed immunoprecipitation of SNAP29 followed by Western blotting with RL2 antibody. The results showed that chemotherapy significantly reduced SNAP29 O‐GlcNAcylation levels compared to untreated controls (Figure 5F,G).

We next sought to identify the O‐GlcNAcylation sites on SNAP29. Prediction using the online database YinOYang suggested that Ser^2^, Ser^61^, Thr^130^, and Ser^153^ of SNAP29 are highly likely to undergo O‐GlcNAcylation (Figure 5H). We then constructed plasmids for wild‐type SNAP29 (SNAP29‐WT), four single‐site mutants (S2A, S61G, T130A, S153G), and a quadruple‐site mutant (S2A/S61G/T130A/S153G, designated SNAP29‐Mut) (Figure S5B,C). IP assays showed that none of the single‐site mutations individually caused a substantial reduction in SNAP29 O‐GlcNAcylation, whereas the quadruple mutant markedly diminished it (Figure 5I), suggesting that Ser^2^, Ser^61^, Thr^130^, and Ser^153^ function cooperatively as key O‐GlcNAcylation sites on SNAP29. To confirm the relationship between these modification sites on SNAP29 and autophagy in GC cells, we stably expressed SNAP29‐WT or SNAP29‐Mut in MKN45 cells. WB analysis showed that, compared to SNAP29‐WT, SNAP29‐Mut increased LC3‐II/LC3‐I ratio and decreased p62 levels, indicating enhanced autophagy (Figure 5J). To confirm these sites are functionally required, we performed rescue experiments. SNAP29 knockdown alone suppressed autophagy, whereas re‐expression of SNAP29‐WT partially restored autophagic flux. Notably, re‐expression of SNAP29‐Mut enhanced autophagy to a greater extent than SNAP29‐WT (Figure S5D). Confocal microscopy analysis of the RFP‐GFP‐LC3 reporter revealed an increase in GFP^−^RFP^+^ puncta (autolysosomes) in SNAP29‐Mut cells compared to SNAP29‐WT cells (Figure 5K,L), demonstrating that SNAP29‐Mut elevated autophagic flux following chemotherapy. CQ treatment further confirmed this conclusion, as SNAP29‐Mut cells displayed a marked increase in GFP^+^RFP^+^ puncta and total LC3 puncta upon CQ treatment (Figure S5E,F). Furthermore, the drug dose‐response curve for SNAP29‐Mut cells showed a marked rightward shift (Figure 5M), indicating significantly reduced drug sensitivity. Similar to the autophagy rescue experiments, knockdown of endogenous SNAP29 enhanced chemosensitivity, whereas re‐expression of SNAP29‐WT or SNAP29‐Mut restored chemoresistance, with SNAP29‐Mut showing a stronger rescue effect (Figure S5G,H). This supports the conclusion that O‐GlcNAcylation at Ser^2^, Ser^61^, Thr^130^, and Ser^153^ of SNAP29 is crucial for autophagy activation and resistance to 5‐FU or OXA. WB further demonstrated that SNAP29‐Mut significantly reduced the levels of cleaved Caspase‐3, cleaved Caspase‐8, and cleaved PARP in MKN45 cells after 5‐FU or OXA treatment (Figure 5N).

In summary, these data demonstrate that OGT specifically interacts with SNAP29 and promotes its O‐GlcNAcylation at Ser^2^, Ser^61^, Thr^130^, and Ser^153^. Inhibition of SNAP29 O‐GlcNAcylation enhances autophagy and reduces 5‐FU or OXA induced cell death in GC.

### 2.6 Inhibition of SNAP29 O‐GlcNAcylation Promotes Chemoresistance by Enhancing SNARE Complex Assembly and Autophagosome Maturation

**FIGURE 6:**
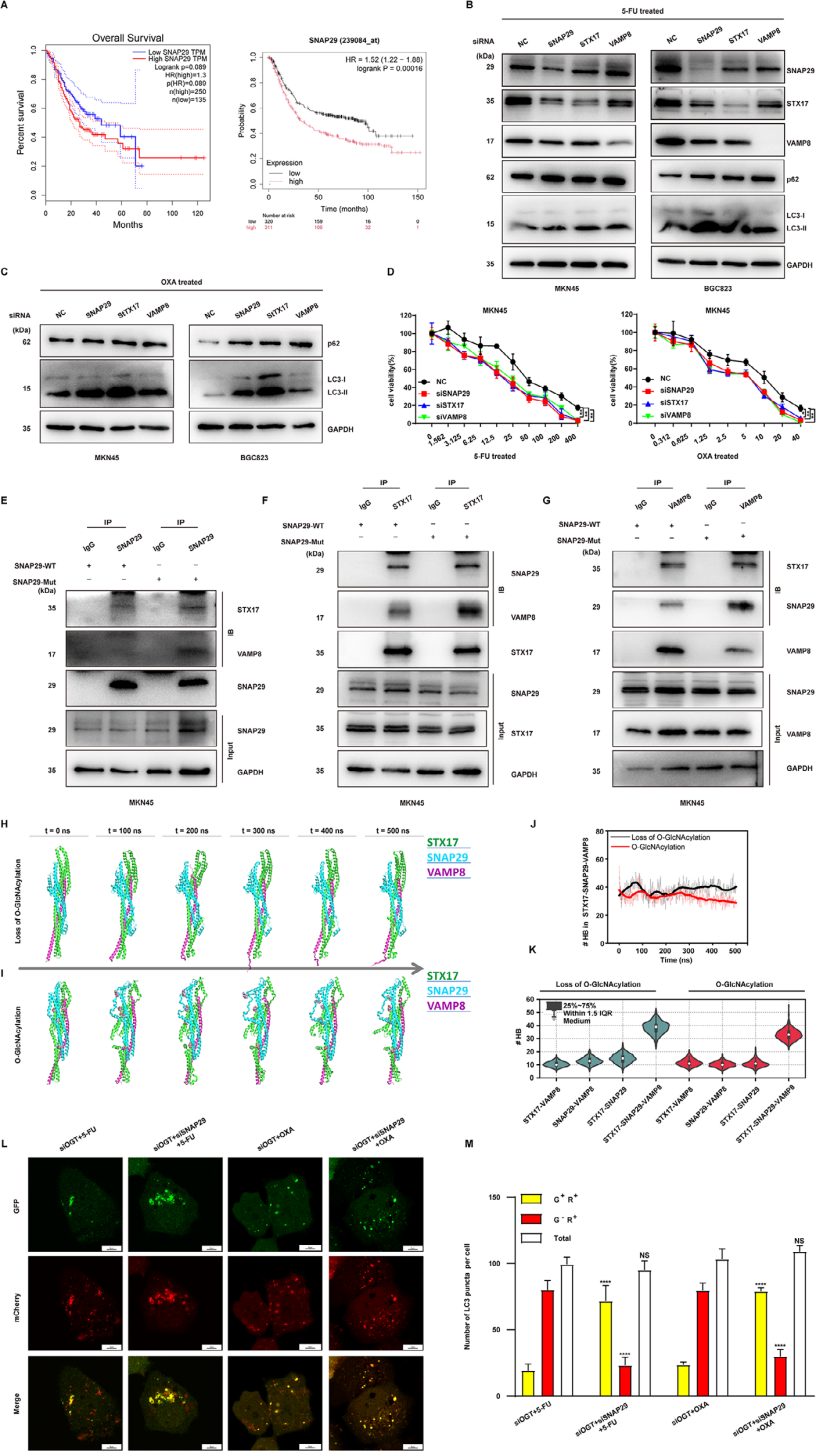
Inhibition of SNAP29 O‐GlcNAcylation Promotes Chemoresistance by Enhancing SNARE Complex Assembly and Autophagosome Maturation. (A) Kaplan–Meier analysis of OS in GC patients from the TCGA database, stratified by SNAP29 expression (left), and Kaplan–Meier curves of OS of patients with GC based on the SNAP29 expression levels(right). (B, C) WB analysis of LC3‐I, LC3‐II, and p62 expression in MKN45 (B) and BGC823 (C) cells transfected with NC, siSNAP29, siSTX17, or siVAMP8 and subsequently treated with 5‐FU or OXA. (D) CCK‐8 assays comparing the viability of MKN45 cells transfected with NC, siSNAP29, siSTX17, or siVAMP8, following treatment with 5‐FU (left) or OXA (right). (E–G) Co‐IP assay in MKN45 cells transfected with SNAP29‐WT or SNAP29‐Mut, using anti‐SNAP29 (E), anti‐STX17 (F), or anti‐VAMP8 (G) antibodies to assess the assembly of the STX17‐SNAP29‐VAMP8 SNARE complex. (H, I) Structural snapshots of the triplex with loss of O‐GlcNAcylation (H) or with O‐GlcNAcylation (I) at 0, 100, 200, 300, 400, and 500 ns. The glycosylated complex exhibits progressive structural drift. (J, K) Inter‐protein hydrogen bond dynamics. The STX17‐VAMP8 interface remained unaffected upon SNAP29 O‐GlcNAcylation. The SNAP29‐VAMP8 and STX17‐SNAP29 interfaces lost approximately 2 and 7 hydrogen bonds, respectively, upon SNAP29 O‐GlcNAcylation, totaling approximately 9 fewer inter‐chain hydrogen bonds. (L, M) Representative fluorescence images of RFP‐GFP‐LC3‐transfected siOGT and siSNAP29 GC cells treated with 5‐FU or OXA (scale: 5 µm, Images were acquired using a 60× objective) (L), and quantitative analysis of GFP^+^RFP^+^, GFP^−^RFP^+^ puncta (M). Data are expressed as mean ± SD of biological replicate experiments. *p < 0.05, **p < 0.01, ****p < 0.0001.

Accumulating evidence indicates that O‐GlcNAcylation regulates protein function, protein‐protein interactions, and enzymatic activity [40, 41]. SNAP29, a component of the Soluble N‐ethylmaleimide‐sensitive factor Attachment protein REceptor (SNARE) complex (SNAP29/STX17/VAMP8), is involved in autophagosome maturation [42, 43]. Data from the TCGA database and Kaplan‐Meier plotter suggest that high expression of SNAP29 is associated with poor prognosis in GC (Figure 6A). Furthermore, IHC analysis of the same 80 paired gastric cancer and adjacent normal tissue samples confirmed that SNAP29 expression was also elevated in gastric cancer tissues (Figure S6A). We next investigated whether SNAP29 O‐GlcNAcylation affects chemoresistance by modulating SNARE complex‐mediated autophagosome maturation in GC cells. First, we examined whether the SNARE complex influences autophagosome maturation in GC following chemotherapy. Correlation analysis in TCGA‐STAD samples revealed significant positive correlations between each member of the SNARE complex and the autophagy marker LC3 (Figure S6B). WB results showed that knockdown of any individual component of the SNARE complex resulted in a concomitant increase in both the LC3‐II/LC3‐I ratio and p62 levels (Figure 6B,C), indicative of blocked autophagosome maturation [44]. We then assessed the impact of this process on chemosensitivity. CCK‐8 assays demonstrated that knockdown of any SNARE complex protein caused a leftward shift in the dose‐response curves to 5‐FU or OXA compared to controls (Figure 6D), indicating increased drug sensitivity upon autophagy inhibition. These results demonstrate that SNARE complex‐mediated autophagosome maturation is crucial for the development of GC chemoresistance. Subsequently, we explored the effect of SNAP29 O‐GlcNAcylation on SNARE complex assembly. Co‐IP assays using anti‐SNAP29 antibody in MKN45 cells transfected with SNAP29‐WT or SNAP29‐Mut plasmids showed that SNAP29‐Mut co‐precipitated more STX17 and VAMP8 protein than SNAP29‐WT (Figure 6E). Conversely, reciprocal Co‐IP experiments using anti‐STX17 or anti‐VAMP8 antibodies also pulled down more SNARE complex proteins in SNAP29‐Mut cells (Figure 6F,G). This indicates that the SNAP29‐Mut facilitates SNARE complex assembly, thereby promoting autophagosome maturation. To gain mechanistic insight into how SNAP29 O‐GlcNAcylation affects SNARE complex assembly, we performed molecular dynamics (MD) simulations comparing SNAP29 with O‐GlcNAcylation (mimicking SNAP29‐WT) and loss of SNAP29 O‐GlcNAcylation (mimicking SNAP29‐Mut) in complex with STX17 and VAMP8. Trajectory snapshots revealed that the complex with loss of SNAP29 O‐GlcNAcylation maintained a compact, well‐ordered four‐helix bundle throughout the 500‐ns simulation (Figure 6H), whereas SNAP29 O‐GlcNAcylation induced progressive structural drift (Figure 6I). RMSD analysis confirmed this divergence: the triplex RMSD of the complex with SNAP29 O‐GlcNAcylation reached substantially higher values, while the complex with loss of SNAP29 O‐GlcNAcylation rapidly equilibrated to a low‐RMSD plateau (Figure S6C). Consistently, RMSD calculated for SNAP29 alone showed that O‐GlcNAcylated SNAP29 deviated more dramatically, indicating that O‐GlcNAcylation directly perturbs SNAP29 conformation (Figure S6D). B‐factor analysis revealed that SNAP29 O‐GlcNAcylation caused markedly elevated B‐factors at the N‐terminus and C‐terminus of SNAP29, while loss of SNAP29 O‐GlcNAcylation displayed uniformly low B‐factors across all chains (Figure S6E). Hydrogen bond analysis revealed that SNAP29 O‐GlcNAcylation reduced inter‐chain hydrogen bonds by approximately 9 in total, with approximately 7 fewer at the STX17‐SNAP29 interface and approximately 2 fewer at the SNAP29‐VAMP8 interface, while the STX17‐VAMP8 interface remained unaffected (Figure 6J,K; Figure S6F–H). Together, these MD simulations reveal that SNAP29 O‐GlcNAcylation destabilizes the four‐helix bundle by disrupting key interfacial hydrogen bonds and increasing conformational flexibility at the SNAP29 termini. To prove that the autophagy activated by OGT knockdown involves SNAP29‐mediated autophagosome maturation, we further knocked down SNAP29 in OGT‐knockdown GC cells. Confocal analysis of the RFP‐GFP‐LC3 reporter showed that, after 5‐FU or OXA treatment, SNAP29 knockdown led to fewer GFP^−^RFP^+^ puncta (autolysosomes) and more GFP^+^RFP^+^ puncta (autophagosomes) compared to the OGT‐knockdown control (Figure 6L,M), indicating inhibition of autophagosome maturation. This demonstrates that SNAP29 knockdown blocks the pro‐maturation effect conferred by OGT knockdown.

In summary, our results indicate that inhibition of SNAP29 O‐GlcNAcylation promotes SNARE complex assembly, leading to enhanced autophagosome maturation and consequently, GC resistance to 5‐FU or OXA.

### 2.7 Elevation of O‐GlcNAcylation Sensitizes GC to 5‐FU or OXA In Vivo

**FIGURE 7:**
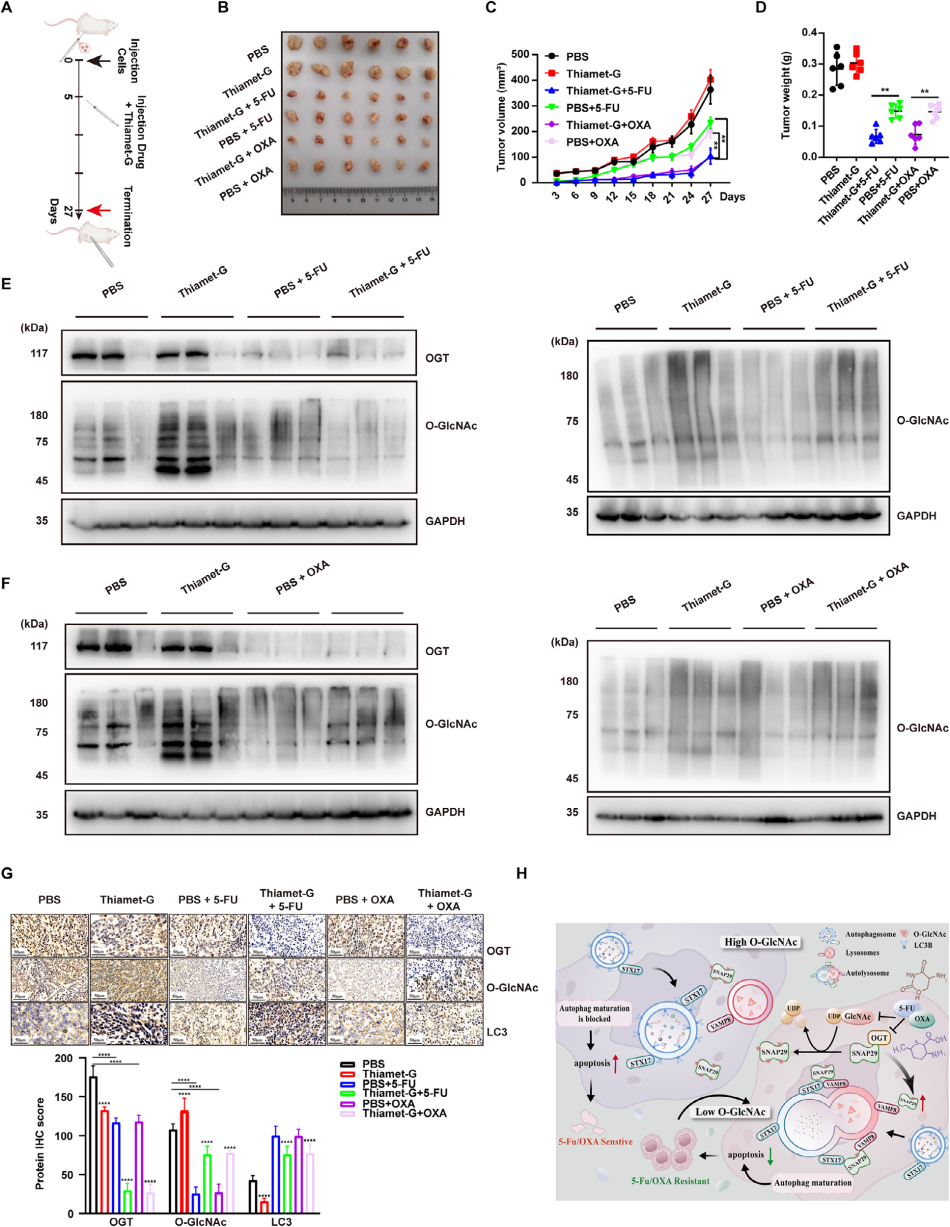
Elevation of O‐GlcNAcylation Sensitizes GC to 5‐FU or OXA In Vivo. (A) Schematic illustration of the MKN45‐line‐derived subcutaneous xenograft mouse model. (B) 1 × 10^6^ MKN45 cells were subcutaneously injected into nude mice (n = 6). Mice were treated for 27 days, and tumors were collected for imaging. (C) Tumor volume was recorded every 3 days. (D) Tumor weight was measured after 27 days. (E, F) WB analysis of O‐GlcNAcylation levels in tumor tissues from the indicated treatment groups. Samples from mice treated with 5‐FU (D), OXA (E), and Thiamet‐G. (G) IHC analysis of OGT, O‐GlcNAcylation and LC3 in tumor sections using indicated antibodies (Scale bars: 50 µm). (H) Model illustrating the proposed mechanism by which chemotherapy‐induced downregulation of OGT disrupts SNAP29 O‐GlcNAcylation, promoting protective autophagy and chemoresistance, which in turn establishes a feedforward loop to perpetuate drug tolerance. Data are expressed as mean ± SD of biological replicate experiments. **p < 0.01.

Finally, we evaluated the impact of modulating O‐GlcNAcylation on the efficacy of 5‐FU or OXA in GC using the small‐molecule activator Thiamet‐G in vivo. A subcutaneous xenograft model was established in nude mice using MKN45 GC cells. Mice were divided into six groups: PBS,Thiamet‐G, Thiamet‐G + 5‐FU, PBS + 5‐FU, Thiamet‐G + OXA, and PBS + OXA. They received daily intraperitoneal injections of the respective agents and were sacrificed one month later to harvest the tumors (Figure 7A). The results showed that Thiamet‐G treatment reduced tumor volume and weight following either 5‐FU or OXA therapy (Figure 7A–D). Tumor tissues from each group were then analyzed. WB and IHC confirmed that the Thiamet‐G groups exhibited higher global O‐GlcNAcylation compared to their respective PBS control groups (Figure 7E–G). Compared to the non‐chemotherapy groups (PBS and Thiamet‐G alone), treatment with 5‐FU or OXA reduced OGT expression and global O‐GlcNAcylation (Figure 7E–G). Importantly, Thiamet‐G treatment decreased intratumoral LC3 levels (Figure 7G). These findings indicate that Thiamet‐G‐induced elevation of O‐GlcNAcylation enhances GC sensitivity to 5‐FU or OXA in vivo, and this sensitization effect is associated with the inhibition of protective autophagy. Given that Thiamet‐G functions by specifically inhibiting OGA, we also assessed the clinical relevance of OGA in GC. We found that high OGA expression in TCGA‐STAD samples was significantly correlated with poorer clinicopathological features, including higher grade, lymph node metastasis, advanced T stage, and TNM stage (Figure S7A). Furthermore, subgroup analysis of these features using the Kaplan‐Meier plotter suggested that high OGA expression was consistently associated with worse survival prognosis (Figure S7B).

In conclusion, these findings demonstrate that targeting SNAP29 O‐GlcNAcylation to regulate autophagy is crucial for GC chemoresistance. Pharmacologically elevating O‐GlcNAcylation with Thiamet‐G sensitizes GC to chemotherapy, suggesting that combining OGA inhibitors with 5‐FU or OXA is a promising therapeutic strategy to overcome chemoresistance.

## 3 Discussion

Compared to surgery alone, perioperative chemotherapy improves the five‐year survival rate of gastric cancer (GC) by 13% and reduces the risk of death by 25% [45]. However, chemoresistance occurs in approximately 30% of GC patients, leading to tumor recurrence [46], which remains a major challenge in GC treatment. Therefore, an in‐depth investigation into the mechanisms of GC chemoresistance is urgently needed. In this study, by integrating clinical samples, in vivo, and in vitro data, we elucidated the sensitizing role of OGT‐mediated O‐GlcNAcylation in GC response to 5‐FU or OXA and revealed a novel mechanism by which OGT inhibition regulates GC chemoresistance.

OGT, the sole transferase catalyzing O‐GlcNAcylation, plays a vital role in maintaining normal cellular physiology [47]. Many cancer‐associated proteins undergo O‐GlcNAcylation, underscoring its critical role in tumor progression and prognosis [48, 49]. While most studies report that OGT and O‐GlcNAcylation are upregulated in various cancers and act as key promoters of tumorigenesis and progression, their roles in cancer drug response have shown inconsistent results [13, 17, 50, 51]. For instance, acute treatment with doxorubicin (DOX) or camptothecin (CPT) activates the HBP, inducing O‐GlcNAcylation, which leads to the activation of pro‐survival signaling pathways and chemoresistance in breast cancer cells [52]. In contrast, DR4 O‐GlcNAcylation enhances TRAIL sensitivity [17]. Another study showed that O‐GlcNAcylation promotes sensitivity to bortezomib in mantle cell lymphoma by enhancing the stability of truncated Bid [53]. These findings suggest that the effect of O‐GlcNAcylation on drug responsiveness varies across different cancers, governed by distinct regulatory mechanisms. Through the integration of clinical tumor specimens, transcriptomic sequencing data, and database information, this study found that high OGT expression was associated with better prognosis in GC patients who received fluoropyrimidine‐ or platinum‐based chemotherapy, but not in those who did not. This suggests that OGT may regulate GC chemosensitivity. Furthermore, GC cell lines with higher OGT expression exhibited lower IC50 values for 5‐FU or OXA. In vivo experiments showed that tumors in the stable OGT‐knockdown group had larger volumes and weights. In vitro rescue experiments further confirmed that re‐expression of OGT in OGT‐knockdown cells restored chemosensitivity, demonstrating the specificity of this effect. Therefore, our results demonstrate that OGT promotes GC chemosensitivity. It is well known that cells undergo c changes to survive under stress, which is a common mechanism for acquired resistance. Previous studies found that acute chemotherapy induces increased O‐GlcNAcylation, contributing to chemoresistance [52]. In contrast, our study revealed that both 5‐FU or OXA treatment suppressed the synthesis of the metabolic substrate UDP‐GlcNAc and downregulated OGT protein expression, thereby inhibiting global O‐GlcNAcylation. This aligns with our finding that OGT knockdown promotes resistance. These results suggest an atypical regulation of the HBP in chemoresistance, characterized by reduced HBP flux during drug stress. Glycolysis is a direct energy source for cells, and the HBP is a branch pathway of glycolysis [54]. Under chemotherapy stress, cells may require increased glycolytic flux to meet energy demands, which could partially explain the observed suppression of the HBP. Previous research indicated that under nutrient deprivation, reduced OGT levels decrease c‐MYC, enabling tumor cells to cope with glucose‐mediated metabolic stress and promoting cancer cell survival [55]. Our findings further confirm that acute 5‐FU or OXA treatment suppresses OGT and its mediated O‐GlcNAcylation in GC cells. Given the complexity of the mechanisms regulating OGT expression and that this is not the central focus of the present study, we have not fully elucidated the precise mechanism. Nevertheless, this suppression reduces chemotherapy‐induced apoptosis under stress, ultimately leading to chemoresistance.

5‐FU metabolites primarily target thymidylate synthase (TS), inhibiting DNA synthesis and leading to cell cycle arrest and death [51]. OXA metabolites form DNA cross‐links, causing DNA damage and subsequent cell death [56]. Our flow cytometry results showed that OGT knockdown or O‐GlcNAcylation inhibition reduced the proportion of apoptotic GC cells after 5‐FU or OXA treatment. Given that these two drugs induce cell death via distinct molecular mechanisms, the precise mechanism by which OGT concurrently regulates sensitivity to both warrants further exploration. Autophagy is widely implicated in cancer multidrug resistance. We found that both 5‐FU and OXA activated autophagy, which promoted GC cell survival against drug toxicity. Previous studies indicate that OGT regulates autophagic activity. For instance, Jin et al. reported that blocking O‐GlcNAcylation induces AMPK‐dependent autophagy in bladder cancer cells [57]. Guo et al. demonstrated that OGT modulates autophagy by mediating the O‐GlcNAcylation of the SNARE protein SNAP29 in C. elegans and HeLa cells [44]. However, the relationship between OGT and autophagy in GC remained unclear. Our analysis of TCGA‐STAD samples revealed that high OGT expression was negatively correlated with multiple autophagy‐related pathways, whereas high expression of OGA, which represents O‐GlcNAcylation inhibition, was associated with autophagy activation. Subsequent in vitro and in vivo experiments confirmed this negative regulatory relationship between O‐GlcNAcylation and autophagy, an effect that was amplified upon 5‐FU or OXA treatment. Nevertheless, whether autophagy mediates the pro‐resistance effect of OGT knockdown was uncertain. Using chloroquine (CQ), a non‐specific autophagy inhibitor commonly employed in research [58, 59], our dose‐response curve assays and flow cytometry apoptosis analysis demonstrated that CQ completely reversed the resistance‐promoting effect of OGT knockdown. In summary, our findings indicate that autophagy activation is a key mechanism by which OGT knockdown promotes resistance to 5‐FU or OXA in GC.

SNAP29 is a cytosolic protein lacking a transmembrane domain. It assembles with STX17 and VAMP8 to form a SNARE complex that mediates autophagosome‐lysosome fusion [60]. Prior studies have focused on the impact of the SNARE complex and its post‐translational modifications on autophagic flux [44, 61, 62]. For example, SNAP29 O‐GlcNAcylation affects SNARE complex assembly [44], but its role in GC treatment and prognosis was unreported. Our study found that OGT specifically interacts with SNAP29. Using site‐directed mutagenesis and functional assays, we demonstrated that mutations at Ser^2^, Ser^61^, Thr^130^, and Ser^153^ of SNAP29 significantly reduced its O‐GlcNAcylation, enhanced autophagy, and promoted GC chemoresistance. Furthermore, rescue experiments showed that re‐expression of SNAP29‐WT partially restored autophagic flux and cell viability in SNAP29‐knockdown cells, while re‐expression of SNAP29‐Mut exhibited a stronger rescue effect, confirming that these sites are functionally required for SNAP29‐mediated autophagy and chemoresistance. By analyzing drug sensitivity following individual knockdown of SNARE complex members and Co‐IP results with SNAP29‐Mut, we further established that the intact SNARE complex is crucial for GC chemoresistance. Molecular dynamics simulations further suggested that SNAP29 O‐GlcNAcylation destabilizes the four‐helix bundle by disrupting inter‐chain hydrogen bonds (approximately 9 fewer hydrogen bonds in total) and increasing conformational flexibility at the SNAP29 termini, providing atomic‐level insight into how this modification inhibits SNARE complex assembly. Moreover, high SNAP29 mRNA expression in GC databases correlated with chemoresistance and poor prognosis. Pellegrini et al. reported that SNAP29 O‐GlcNAcylation blocks autophagosome‐lysosome fusion and promotes apoptosis via reactive oxygen species generation [63]. This aligns with our findings, as SNAP29‐Mut cells expressed lower levels of apoptotic proteins: cleaved Caspase‐3, cleaved Caspase‐8, and cleaved PARP. Collectively, our results indicate that SNAP29 and its O‐GlcNAcylation‐mediated regulation of the SNARE complex confer sensitizing functions in GC during 5‐FU or OXA therapy.

More importantly, our in vivo experiments showed that combining the OGA inhibitor Thiamet‐G with 5‐FU or OXA significantly enhanced chemosensitivity. Two other OGA inhibitors, MK‐8719 and ASN120290, have been recognized by the US Food and Drug Administration for treating progressive supranuclear palsy, a neurodegenerative tauopathy [64]. Furthermore, several inhibitors targeting HBP enzymes, OGT, and OGA have shown potential in anti‐cancer therapy [65]. Therefore, our study provides a rationale for exploring combination chemotherapy strategies targeting O‐GlcNAcylation. This study has certain limitations. We have not yet collected tumor tissue samples from GC patients with clinical chemoresistance to validate OGT expression levels. Additionally, this study primarily used acute drug treatment models without establishing long‐term chemoresistant gastric cancer cell lines. In future studies, we will collect clinical chemoresistant specimens and generate resistant cell models to more comprehensively elucidate the role of OGT in chemoresistance. In conclusion, this study reveals a previously unknown epigenetic regulatory mechanism: 5‐FU or OXA reduces global O‐GlcNAcylation in GC and downregulation of SNAP29 O‐GlcNAcylation promotes GC cell survival by enhancing chemotherapy‐induced protective autophagy. OGT specifically interacts with and promotes O‐GlcNAcylation of SNAP29. Mutations at Ser^2^, Ser^61^, Thr^130^, and Ser^153^ of SNAP29 significantly reduce its O‐GlcNAcylation, which facilitates the assembly of the STX17‐SNAP29‐VAMP8 SNARE complex, leading to enhanced autophagy and ultimately increased tolerance of GC cells to the toxicity of 5‐FU or OXA chemotherapy. This finding expands our understanding of the critical role of SNAP29 O‐GlcNAcylation in GC chemoresistance and offers a potential novel therapeutic strategy to enhance the efficacy of 5‐FU or OXA in GC treatment.

## 4 Experimental Section

### 4.1 Patients and Tissue Samples

The 80 paired gastric cancer and adjacent normal tissue pathological sections were obtained from the Department of Pathology, The First Affiliated Hospital of Sun Yat‐sen University. Eight paired GC tissue samples, each pair comprising tumor tissue and adjacent non‐tumor tissue from the same patient, were obtained from patients undergoing radical resection in the Department of Gastrointestinal Surgery, The First Affiliated Hospital of Sun Yat‐sen University (Guangzhou, Guangdong, China). No neoadjuvant chemotherapy was administered to any patient prior to surgery, and all postoperative specimens were pathologically confirmed as gastric adenocarcinoma. The standard protocol involved immediate snap‐freezing of all resected tissues in liquid nitrogen, with subsequent storage at −80°C until required for analysis. Depending on experimental requirements, each paired sample was subjected to Western blot (WB), quantitative polymerase chain reaction (qPCR), and/or immunohistochemistry (IHC) assays. All participants provided written informed consent under a protocol approved by the Ethics Committee of The First Affiliated Hospital of Sun Yat‐sen University, and all human tissue procedures were performed in compliance with institutional, national, and Declaration of Helsinki ethical standards.

### 4.2 Cell Lines and Culture

The BGC823, MKN45, AGS, and MKN1 human GC cell lines were provided by the Chinese Academy of Sciences Cell Bank (Shanghai, China). The GC cell lines were maintained at 37°C with 5% CO_2_ in RPMI‐1640 (Gibco, USA) medium containing 1% penicillin–streptomycin and 10% fetal bovine serum (FBS; Gibco, USA). The GC cell lines were routinely authenticated and verified to be free from mycoplasma contamination. Unless otherwise indicated in the figure legends, the following drug concentrations were used: 5‐FU (10–50 µM), OXA (1–10 µM), Thiamet‐G (0–10 µM), OSMI‐1 (50 µM), Z‐VAD‐FMK (5 µM), 3‐methyladenine (3‐MA, 5 mM), and chloroquine (CQ, 10 µM). Briefly, cells were grown in 6‐well or 96‐well plates to the desired density and allowed to reach 60%–70% confluence before treatment with the indicated compounds. Subsequent analyses were performed after 24–48 h of drug exposure.

### 4.3 Animal Studies

All animal studies adhered to the National Institutes of Health (NIH) guidelines and were approved by the Animal Ethics Committee of The First Affiliated Hospital of Sun Yat‐sen University. To induce subcutaneous tumors, 4–5‐week‐old nude mice received injections of 1 × 10^6^ MKN45 cells into the left flank. These included wild‐type, negative control (NC), and OGT‐knockdown (shOGT) cells, each suspended in 100 µL phosphate‐buffered saline (PBS). All nude mice were supplied by the Animal Center of the First Affiliated Hospital of Sun Yat‐sen University. Each experimental group consisted of six mice. Beginning 3–6 days post‐inoculation, mice were administered daily intraperitoneal injections according to their assigned groups: 5‐FU (4 mg/kg/day), OXA (0.5 mg/kg/day), Thiamet‐G (20 mg/kg/day), or an equal volume of PBS (control). Tumor volume was determined by the formula V = π × (d^2^ × D) / 6, where d and D denote the minor and major tumor axes, respectively. Measurements were recorded at three‐day intervals. Tumor growth was monitored until day 27. Termination of the experiment occurred on day 28, at which point all mice underwent euthanasia and subsequent collection of tumor tissues for weighing and imaging. Portions of each tumor were fixed in formalin for paraffin embedding, while the remaining tissues were snap‐frozen at −80 °C for later use.

### 4.4 Vector Construction and Transfection

Complementary DNAs (cDNAs) encoding OGT, SNAP29, and its single‐site mutant form SNAP29 (S2A,S61G,T130A,S153G) and quadruple‐site mutant form SNAP29 (S2A/S61G/T130A/S153G) were cloned into pcDNA3.1 vectors. Transfection was performed following the seeding of 3 × 10^5^ cells per well in 6‐well plates. A mixture of 2 µg plasmid DNA and 2 µL Neofect transfection reagent (China) was used for each well. Small interfering RNAs (siRNAs) targeting OGT, SNAP29, STX17, VAMP8, as well as NC siRNAs, were obtained from Jicheng Gene Technology (Guangzhou, China). Transfection of siRNAs was carried out with Lipofectamine 3000 reagent (Invitrogen, USA) for 72 h in accordance with the manufacturer's instructions. Stable cell lines were generated using a standard plating method. Specifically, cells were seeded onto coverglasses in 6‐well plates and cultured for 24 h to achieve 70% confluence prior to transfection. cDNAs encoding RFP‐GFP‐LC3 were cloned into the lentiviral vector pLenti (Dahong Biotechnology, Guangzhou, China), while OGT‐shRNA and shNC were cloned into the lentiviral vector GVYB104 (Jicheng Gene Technology, Guangzhou, China). Viral particles were produced and used for infection according to standard procedures. Stable transfections were selected with the corresponding antibiotics for 2 weeks before subsequent experiments.The siRNA sequences used in this study were as follows:

siOGT#1, 5ʹ‐GCCAUCGUAUACUGUAACUTT‐3ʹ; siOGT#2, 5ʹ‐GUUGGCACAUCGAGAAUAUTT‐3ʹ; siOGT#3, 5ʹ‐CCUGGCUUGUGUAUACUAUTT‐3ʹ; siSNAP29, 5ʹ‐AGACAGAAAUUGAGGAGCATT‐3ʹ; siSTX17, 5ʹ‐CCGAAAGGAUGACCUAGUATT‐3ʹ; siVAMP8,5ʹ‐GCAACAAGACAGAGGAUCUTT‐3ʹ.

### 4.5 Western Blotting

Cell and tissue samples were subjected to total protein extraction using a previously described protocol [66]. Briefly, following a 1‐h blocking step, the membranes were subjected to an overnight incubation with primary antibodies (Table S1) at 4°C under constant agitation. After washing in TBST, the membranes were probed for 1 h at room temperature with horseradish peroxidase (HRP)‐conjugated secondary antibodies (Table S1). Signals from the protein bands were generated using a hypersensitive chemiluminescence substrate (WBKLS0500, Merck Millipore).

### 4.6 RNA Extraction and Quantitative Real‐Time PCR

Cell and tissue samples were subjected to total RNA extraction using the RNA‐Quick Purification Kit (RN001, Yesen Biotechnology, Shanghai, China). Following cDNA synthesis with the Evo M‐MLV RT Premix (AG11706, AG Bio) as per the manufacturer's protocol, qPCR was carried out using the SYBR Green Premix Pro Tag HS qPCR Kit II (AG11702, AG Bio). All primer sequences used in this study are listed in TableS1.

### 4.7 Immunohistochemistry (IHC)

Following deparaffinization and rehydration of the tissue sections, antigen retrieval was conducted in 0.01 mol/L ethylenediaminetetraacetic acid (EDTA) (pH 8.0). Inhibition of endogenous peroxidase was achieved through a 10‐minute incubation in 3.0% hydrogen peroxide. Nonspecific binding was prevented by blocking with 5% goat serum containing 0.25% Triton X‐100 for 40 min. At 4°C overnight, the sections were exposed to the respective primary antibodies (Table S1). Following washes, HRP‐conjugated secondary antibodies were applied, and signals were detected using 3,3′‐diaminobenzidine (DAB) as the chromogenic substrate. Finally, counterstaining was performed using hematoxylin, and the sections were examined by light microscopy.

### 4.8 Immunofluorescence (IF)

Cells seeded on coverslips underwent fixation (4% PFA, 15 min), permeabilization (0.25% Triton X‐100, 20 min, RT), and blocking (5% goat serum, 40 min). At 4°C overnight, the coverslips were exposed to the corresponding primary antibodies at optimal dilutions (Table S1). Following washes, the samples were labeled with fluorophore‐conjugated secondary antibodies and DAPI for nuclear visualization under light‐protected conditions. Fluorescence microscopy imaging was performed after mounting the coverslips with anti‐fade medium.

### 4.9 Cell Viability Assays

Cell seeding was performed by uniformly distributing GC cells into 96‐well plates (5 × 10^3^ cells per well) followed by a 24 h incubation at 37°C with 5% CO_2_. Treatment consisted of exposing the GC cells to gradient concentrations of 5‐FU or OXA for 48 h. Cell viability was determined using the Cell Counting Kit‐8 (CCK‐8; KG10001, GlpBio, USA) per the manufacturer's instructions. After 2 h incubation, absorbance was measured at 450 nm with a microplate reader.

### 4.10 Apoptosis Assay

To evaluate apoptosis in vitro, cells were plated in 6‐well plates to reach approximately 70% confluence on the following day. Cells were exposed separately to PBS, 5‑FU, or OXA. After treatment, they were digested with EDTA‑free trypsin, pelleted by centrifugation at 1000 ×*g* for 5 min, and washed twice with PBS. For staining, the cell pellets underwent resuspension in 500 µL binding buffer and successive incubation with 5 µL Annexin V–allophycocyanin (APC) and 5 µL propidium iodide (PI) (KGA1107‐100; KeyGEN). Flow cytometric analysis was performed within 1 h after 10 min of dark incubation.

### 4.11 Transmission Electron Microscopy (TEM)

For the preparation of ultrathin sections, control and si‐OGT‐transfected BGC823 cells were exposed to 5‐FU or OXA. After initial fixation for 24 h at room temperature with 2.5% glutaraldehyde in phosphate buffer, the samples underwent secondary fixation in 1% osmium tetroxide for 2 h. After fixation, dehydration was performed through a graded ethanol series, followed by resin embedding. Ultrathin sections (60–80 nm) underwent mounting on copper grids and staining with 2% uranyl acetate plus lead citrate. Observation was carried out with a transmission electron microscope (JEM‐1400) at an accelerating voltage of 80 kV, and images were captured for analysis.

### 4.12 Nano‐LC–MS/MS Analysis

For proteomic analysis, peptides were separated using an EASY‐nLC 1200 system coupled online to a Q Exactive HF‐X mass spectrometer (Thermo Fisher Scientific, USA). Samples, reconstituted in 0.1% formic acid (Solvent A), were first loaded onto a self‐packed trap column (100 µm × 2 cm, 3 µm C18) and then separated on an analytical column (150 µm × 15 cm, 1.9 µm C18) with a 75‐min linear gradient from 4% to 100% Solvent B (0.1% FA in 80% ACN) at 600 nL/min. Eluting peptides were electrosprayed at 2.0 kV. MS1 spectra (m/z 300–1400) were acquired at 120,000 resolution (AGC 3E6, max IT 80 ms). Up to 60 most intense precursors were selected for HCD fragmentation (NCE 27%), and MS2 spectra were recorded at 7,500 resolution (AGC 5E4, max IT 20 ms) with a 12‐s dynamic exclusion window.

### 4.13 Non‐Targeted Metabolomics Analysis

Samples were extracted with pre‑cooled methanol/acetonitrile/water (2:2:1, v/v/v), vortexed, ultrasonicated at low temperature for 30 min, incubated at −20°C for 10 min, and centrifuged (14 000 g, 4°C, 20 min). The supernatant was dried, reconstituted in acetonitrile/water (1:1, v/v), and centrifuged again before LC‑MS analysis. Separation was performed on a HILIC column (ACQUITY UPLC BEH Amide, 1.7 µm, 2.1 × 100 mm) with a gradient of water containing 25 mM ammonium acetate/ammonia (A) and acetonitrile (B) at 0.5 mL/min and 25°C. MS detection was conducted on an AB Triple TOF 6600 or Thermo Orbitrap Exploris 480 system in both ESI positive/negative modes. Quality control samples were included throughout the run.

### 4.14 Autophagic Flux Analysis

As previously described, a BGC823 GC cell line stably expressing the dual‐fluorescence reporter RFP‐GFP‐LC3 was used to monitor autophagic flux. To assess LC3 puncta formation, control and experimental groups were prepared by knocking down OGT or SNAP29, with or without exposure to 5‐FU or OXA. Confocal microscopy was employed to capture fluorescence images. For each group, five randomly selected fields of view were analyzed to quantify red fluorescent protein (RFP) and green fluorescent protein (GFP) puncta, with a minimum of 50 cells counted per group.

### 4.15 Coimmunoprecipitation

Cell lysis was carried out in IP lysis buffer (P0013, Beyotime) supplemented with a 1:50 dilution of phosphatase and protease inhibitor cocktail (P1045, Beyotime). Following centrifugation of the lysates (12 000 rpm, 15 min, 4°C), the cleared supernatants were subjected to overnight incubation at 4°C with coimmunoprecipitation (Co‐IP) grade antibodies under gentle rotation. The following day, Protein A/G magnetic beads (88803, Thermo Fisher) were introduced, with further incubation for 3–4 h at room temperature under constant rotation. After six washes with wash buffer, the bound complexes were eluted by adding 2× SDS‐PAGE loading buffer (LT101, EpiZyme) and heating at 95°C for 10 min, followed by immunoblotting analysis. Detailed information on antibodies and suppliers is listed in Table S1.

### 4.16 Molecular Dynamics Simulations

The structure of human SNAP29 (UniProt: O95721) was predicted using SeedFold. Four O‐GlcNAcylation sites (Ser2, Ser61, Thr130, Ser153) were modified by attaching GlcNAc moieties via CHARMM‐GUI. Each system was solvated in a TIP3P water box with 150 mM NaCl, using CHARMM36m force field for protein and CHARMM36 carbohydrate force field for GlcNAc. Simulations were performed using GROMACS v2022.5 in the NPT ensemble for 500 ns at 303.15 K and 1.0 bar. RMSD, B‐factors, and inter‐chain hydrogen bonds were calculated using GROMACS built‐in tools.

### 4.17 Statistical Analysis

All data were expressed as mean ± SD from at least three independent duplications. Statistical analyses were conducted using GraphPad Prism version 9.0 (GraphPad Software, USA). Differences between two groups were assessed with Student's t‐test, whereas multi‐group comparisons employed one‐way ANOVA with appropriate post hoc analysis. Analysis of nonparametric data involved the Mann–Whitney U test. Prognostic relevance was estimated in univariate analysis using the Kaplan–Meier method. Associations between variables were evaluated by Spearman's rank correlation analysis. A *p*‐value < 0.05 was considered statistically significant.

## Author Contributions

L. T., S. Z., and Z. S. contributed equally to this work. L. T. contributed to Writing – review and editing, Data curation, Conceptualization, Methodology, Validation, Investigation, Software, Project administration, Formal analysis, Resources, and Writing – original draft. S. Z. contributed to Software, Conceptualization, Data curation, Formal analysis, Visualization, Validation, and Investigation. Z. S. contributed to Software, Methodology, Investigation, Project administration, and Formal analysis. T. L. contributed to Resources and Investigation. B. P. contributed to Resources and Investigation. Y. L. contributed to Resources and Investigation. J. Y. contributed to Resources and Investigation. J. P. contributed to Resources and Investigation. K. S. contributed to Investigation, Validation, Writing – review and editing, Supervision, Methodology, Funding acquisition. J. X. contributed to Writing – original draft, Conceptualization, Supervision, Visualization, Validation, Software, Project administration, Formal analysis, and Funding acquisition.

## Funding

This work was supported by the National Natural Science Foundation of China (No. 82473190, No. 82273222, and No. 82203642) and the Natural Science Foundation of Guangdong Province, China (2022A1515012140).

## Conflicts of Interest

The authors declare no conflicts of interest.

## Supporting information




**Supporting File 1**: advs76730‐sup‐0001‐SuppMat.docx.

**Supporting File 2**: advs76730‐sup‐0002‐FigureS1‐S7.zip.

**Supporting File 3**: advs76730‐sup‐0003‐TableS1.docx.

## Data Availability

The data that support the findings of this study are available from the corresponding author upon reasonable request.
