# Supplementary material for: O‐GlcNAcylation Regulation of SNAP29‐Dependent Autophagy Activation Dictates Chemoresistance in Gastric Cancer
Source: Adv Sci (Weinh). 2026 Jul 23:e76730. Online ahead of print. doi: 10.1002/advs.76730 (PMC13393265; doi:10.1002/advs.76730)
Supplement: Supplementary file 1 — Supporting File 1: advs76730‐sup‐0001‐SuppMat.docx. [file ADVS-9999-e76730-s002.docx]

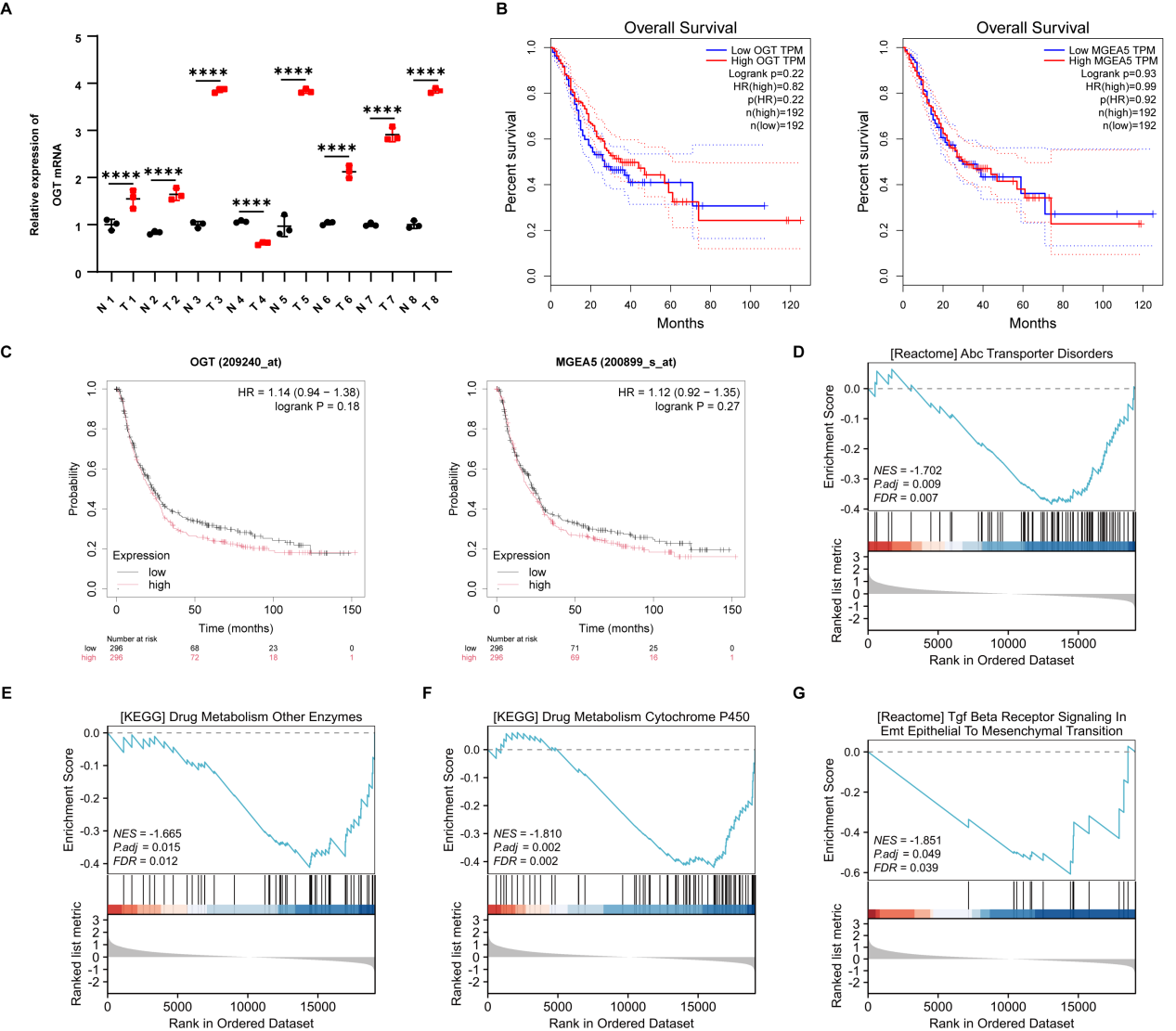


**Figure S1.** High OGT Expression is Inversely Associated with Drug Metabolism, Drug Efflux, and EMT. **A)** Relative expression levels of OGT mRNA in eight pairs of GC and adjacent tissues. **B)** Kaplan-Meier analysis of OS in GC patients from the TCGA database, stratified by OGT (left) and OGA (right)expression. **C)** Kaplan–Meier curves of OS of patients with GC based on the OGT and OGA expression levels. **D)** GSEA reveals negative correlation between high OGT expression in GC from the TCGA database and the Abc Transporter Disorders. **E-G)** GSEA reveals negative correlation between high OGT expression in GC from the TCGA database and the Drug Metabolism Other Enzymes (E), Drug Metabolism Cytochrome P450 (F), Tgf Beta Receptor Signaling In Emt Epithelial To Mesenchymal Transition (G). Data are expressed as mean ± SD of biological replicate experiments. ****p < 0.0001.


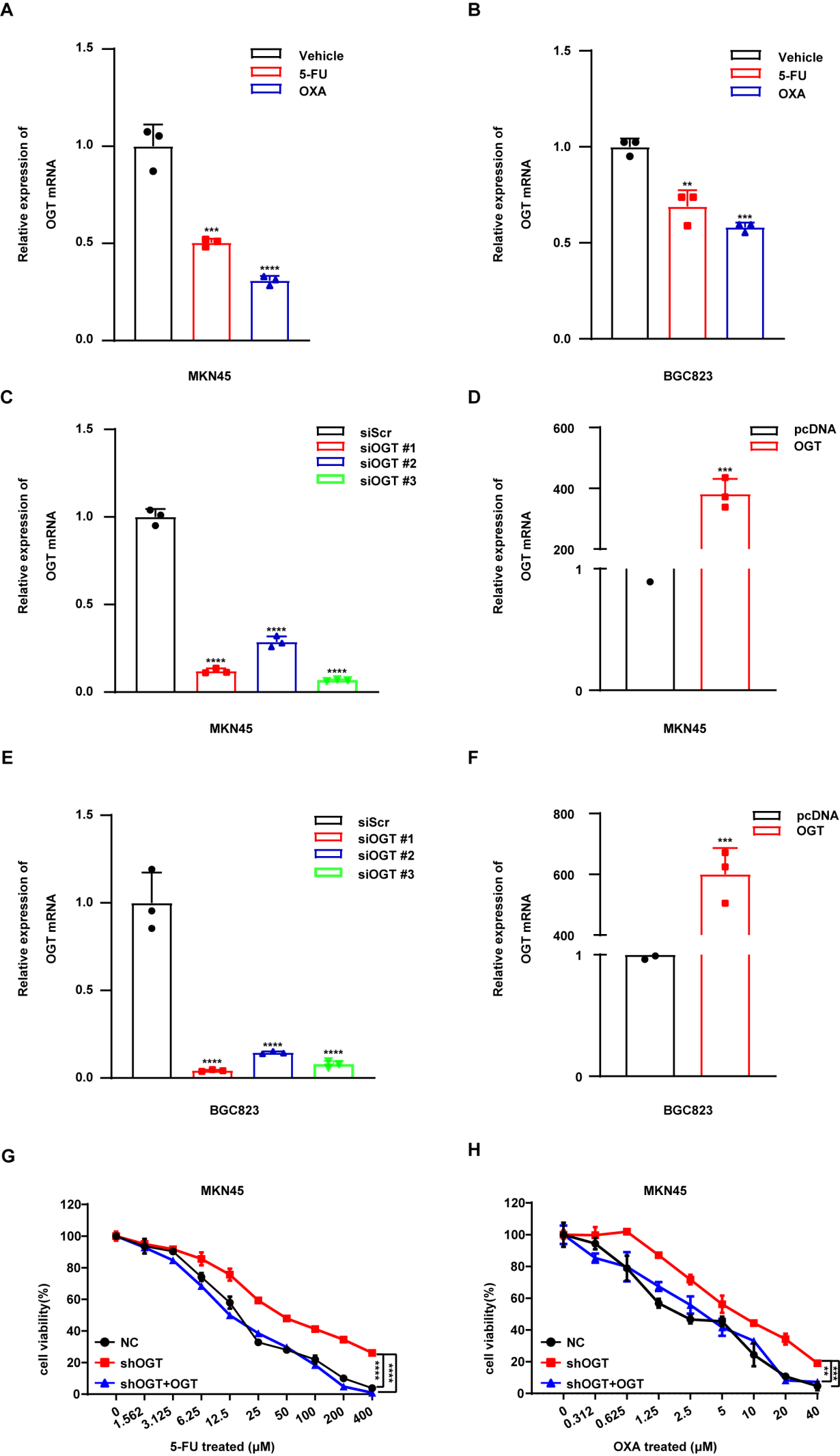


**Figure S2.** 5-FU or OXA Inhibit OGT Expression in GC Cells. **A,B)** Relative mRNA expression levels of OGT in MKN45 (A) and BGC823 (B) cells following treatment with 5-FU (A) or OXA (B). **C,D)** The efficiency of OGT knockdown (C) and overexpression (D) was verified in MKN45 cells by q-PCR. **E,F)** The efficiency of OGT knockdown (E) and overexpression (F) was verified in BGC823 cells by q-PCR. **G,H)** CCK-8 assays assessing the viability of OGT-knockdown MKN45 cells transfected with OGT-overexpressing vectors. Cells were treated with of 5-FU (G) or OXA (H) for 48 hours. Data are expressed as mean ± SD of biological replicate experiments. **p < 0.01, ***p < 0.001, ****p < 0.0001.


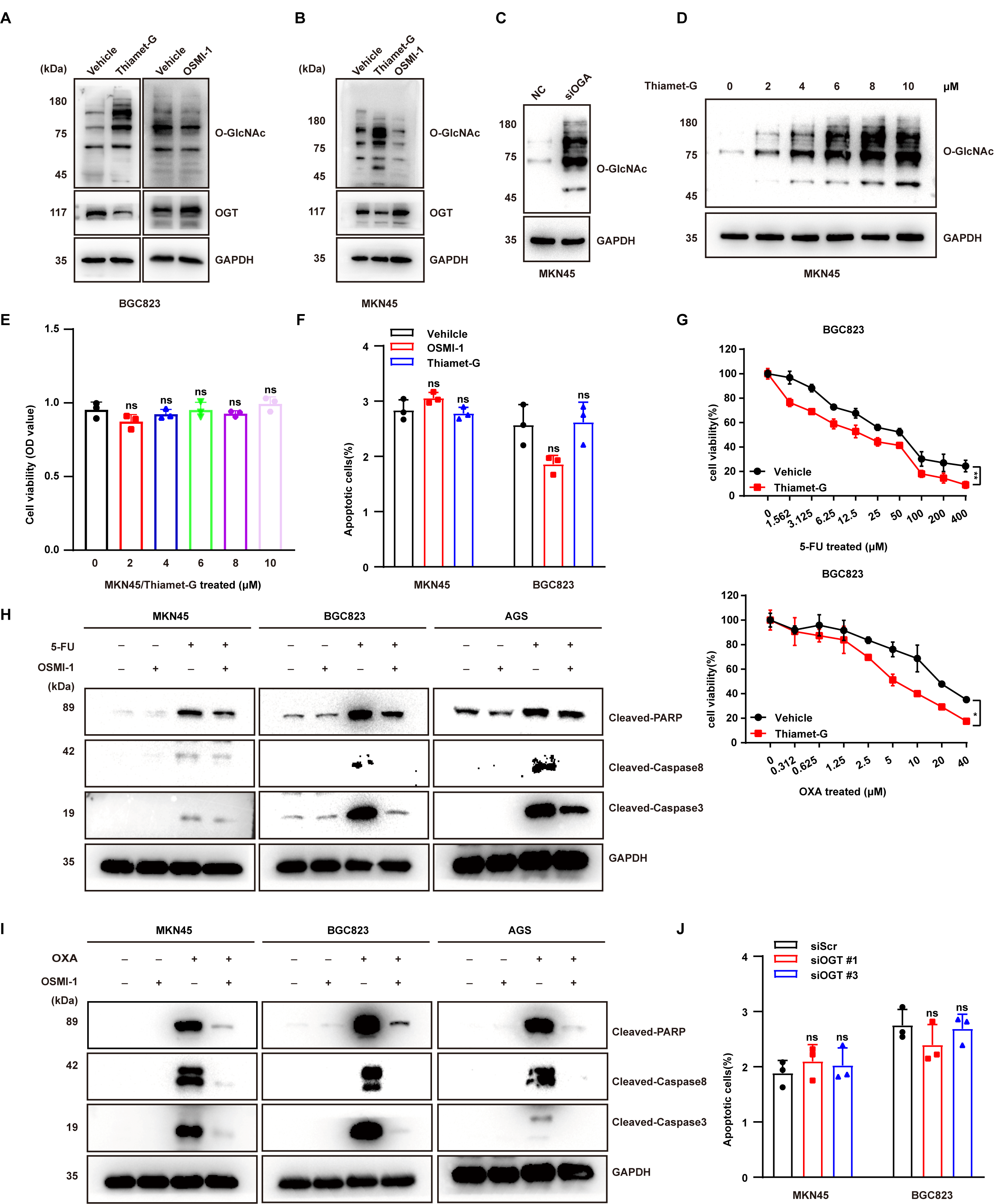


**Figure S3.** Inhibition of O-GlcNAcylation Reduces 5-FU or OXA induced Apoptosis in GC Cells. **A,B)** The efficiency of Thiamet-G and OSMI-1 was verified in BGC823 (A) and MKN45 (B) cells by WB analysis. **C)** Western blot analysis of global O-GlcNAc levels in control and transient OGA knockdown (siOGA) MKN45 cells. **D)** Western blot analysis of global O-GlcNAc levels in MKN45 cells treated with increasing concentrations of Thiamet-G. **E)** CCK-8 assay assessing cell viability of MKN45 cells treated with increasing concentrations of Thiamet-G. **F)** Analysis of apoptosis by flow cytometry. MKN45 and BGC823 cells were treated with vehicle, OSMI-1, or Thiamet-G. Apoptosis was assessed using APC-Annexin V and PI staining. quantitative analysis of apoptotic cells under the indicated conditions. **G)** CCK-8 assays comparing the viability of BGC823 cells treated with vehicle or Thiamet-G, following exposure to increasing concentrations of 5-FU or OXA. **H,I)** MKN45, BGC823 and AGS cells were exposed to 5-FU/OXA with or without OSMI-1. Apoptosis markers, including cleaved Caspase-3, cleaved Caspase-8 and cleaved PARP, levels were examined by WB. **J)** Analysis of apoptosis by flow cytometry. MKN45 and BGC823 cells were transfected with NC or siOGT. Apoptosis was assessed using APC-Annexin V and PI staining. quantitative analysis of apoptotic cells under the indicated conditions. Data are expressed as mean ± SD of biological replicate experiments. *p < 0.05, **p < 0.01.


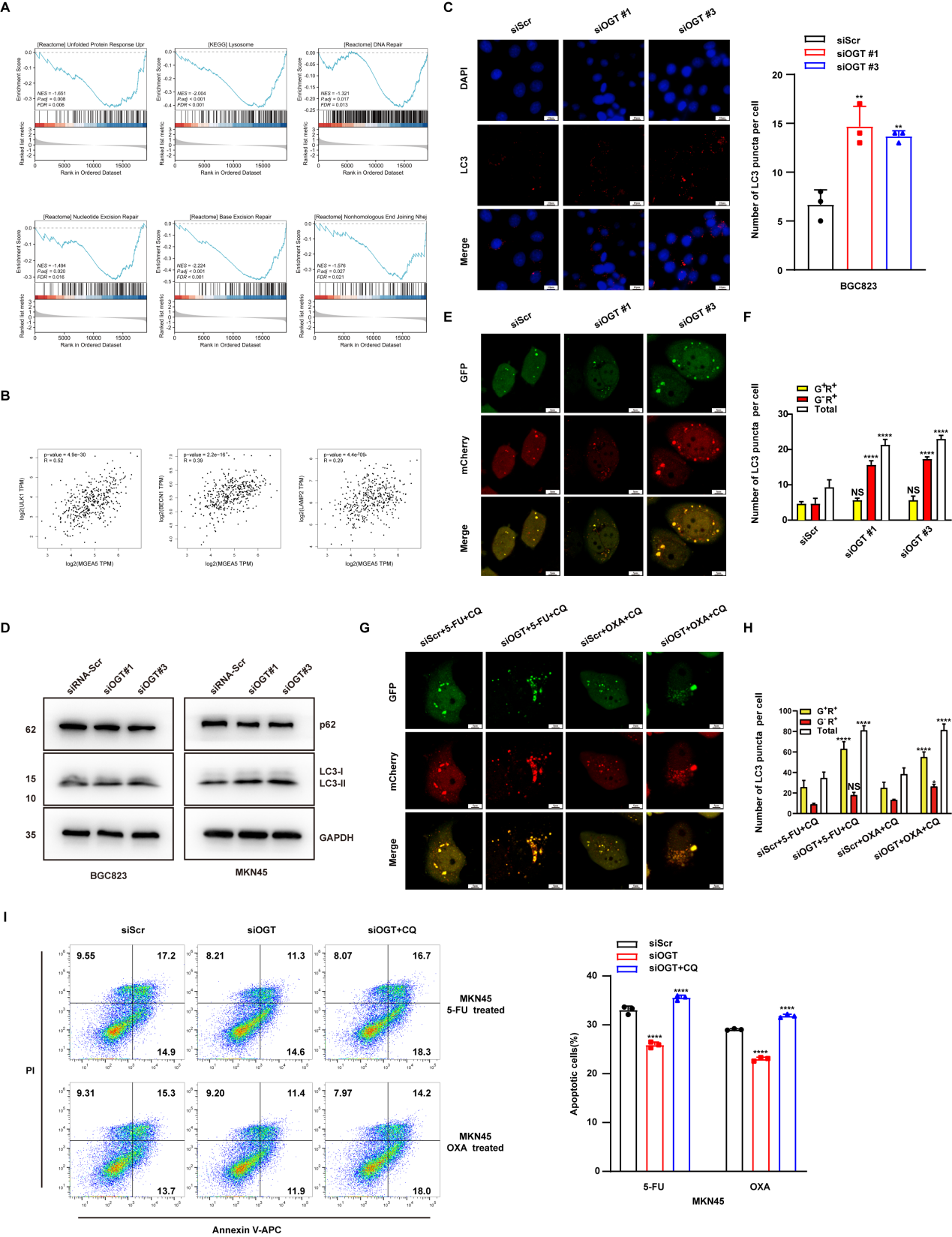


**Figure S4.** OGT Knockdown Inhibits the Basal Autophagy Level in GC Cells. **A)** GSEA reveals negative correlation between high OGT expression in GC from the TCGA database and gene sets related to autophagy as well as chemoresistance associated pathways. **B)** Correlation analysis between OGA expression and ULK1, BECN1, and LAMP2 expression in GC from the TCGA database. **C)** Representative IF images (left) and quantification (right) of LC3 puncta in BGC823 cells transfected with NC or siOGT (Scale bars = 10 μm, Images were acquired using a 40× objective). **D)** WB analysis of LC3-I, LC3-II, and p62 in BGC823 (left) and MKN45 (right) cells transfected with NC or siOGT. **E,F)** Representative fluorescence images of RFP-GFP-LC3-transfected NC and siOGT BGC823 cells (scale: 5 μm, Images were acquired using a 60× objective) (E), and quantitative analysis of GFP⁺RFP⁺, GFP⁻RFP⁺ puncta (F). **G,H)** Representative fluorescence images (scale: 5 μm, Images were acquired using a 60× objective) (G) and quantitative analysis (H) of RFP-GFP-LC3 puncta in NC and siOGT BGC823 cells treated with CQ. **I)** Analysis of apoptosis by flow cytometry. MKN45 cells were transfected with control, siOGT, or siOGT+CQ following exposure to 5-FU or OXA. Apoptosis was assessed using APC-Annexin V and PI staining. Representative flow cytometry plots (left) and quantitative analysis (right) of apoptotic cells under the indicated conditions. Data are expressed as mean ± SD of biological replicate experiments. *p < 0.05, **p < 0.01, ****p < 0.0001.


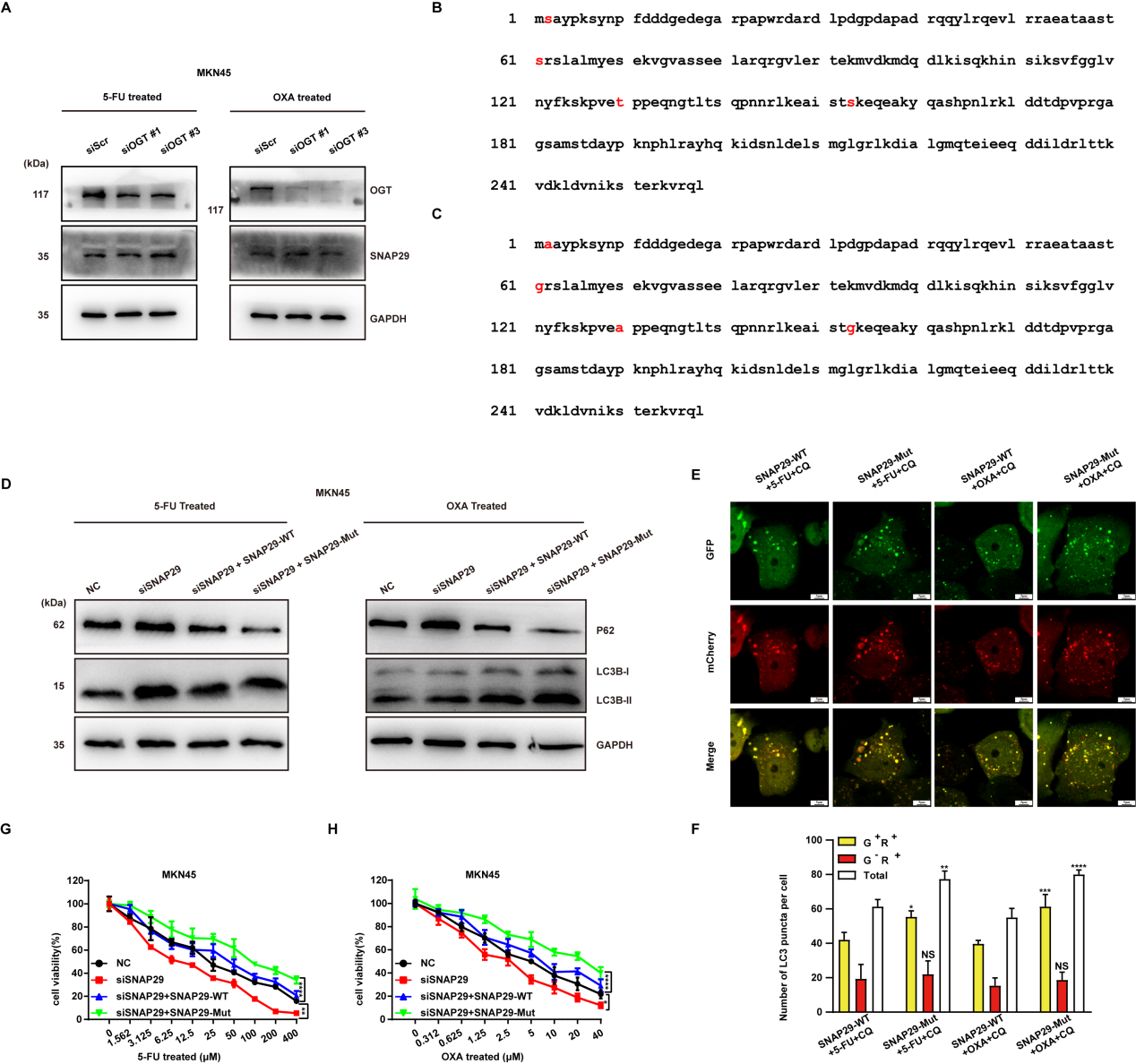


**Figure S5.** Amino Acid Sequence of SNAP29 and Construction of SNAP29-Mut. **A)** WB analysis of SNAP29 protein levels in MKN45 cells transfected with NC or siOGT and subsequently treated with 5-FU or OXA. **B,C)** Amino acid sequences of SNAP29-WT (B) and SNAP29-Mut (C). **D)** WB analysis of LC3-I, LC3-II, and p62 in MKN45 cells transfected with NC or siSNAP29, followed by re-expression of SNAP29-WT or SNAP29-Mut. **E,F)** Representative fluorescence images of RFP-GFP-LC3-transfected SNAP29-WT and SNAP29-Mut GC cells treated with 5-FU or OXA in the presence of CQ (scale bar: 5 μm, Images were acquired using a 60× objective) (E), and quantitative analysis of GFP⁺RFP⁺, GFP⁻RFP⁺ puncta (F). **G,H)** CCK-8 assays comparing the viability of MKN45 cells transfected with NC or siSNAP29 and re-expressed with SNAP29-WT or SNAP29-Mut, following treatment with 5-FU (G) or OXA (H). Data are expressed as mean ± SD of biological replicate experiments. *p < 0.05, **p < 0.01, ***p < 0.001, ****p < 0.0001.


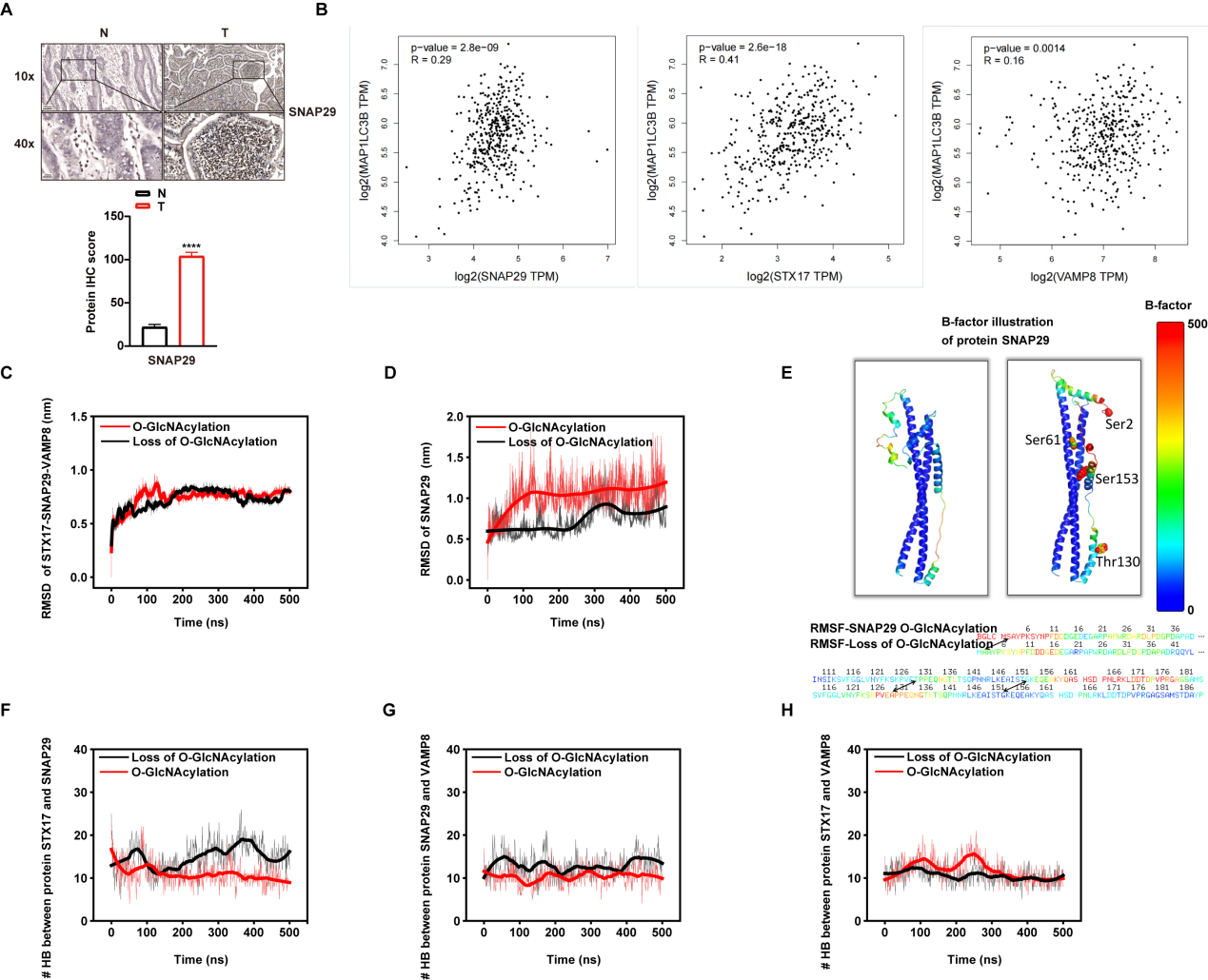


**Figure S6.** SNARE Complex is Significantly Positively Correlated with the LC3. **A)** Representative IHC images of SNAP29 in GC and adjacent tissues. Scale bars: 25μm-100 μm. **B)** Correlation analysis between LC3 expression and SNAP29, STX17, and VAMP8 expression in GC from the TCGA database. **C,D)** RMSD trajectories of the triplex (C) and SNAP29 alone (D). **E)** B-factor profiles of SNAP29. Loss of SNAP29 O-GlcNAcylation displays uniformly low B-factors across all residues. In contrast, SNAP29 O-GlcNAcylation causes markedly elevated B-factors at the N-terminus and C-terminus. **F-H)** Inter-protein hydrogen bond dynamics. The STX17-VAMP8 interface remained unaffected upon SNAP29 O-GlcNAcylation. The SNAP29-VAMP8 and STX17-SNAP29 interfaces lost approximately 2 and 7 hydrogen bonds. Data are expressed as mean ± SD of biological replicate experiments. ****p < 0.0001.


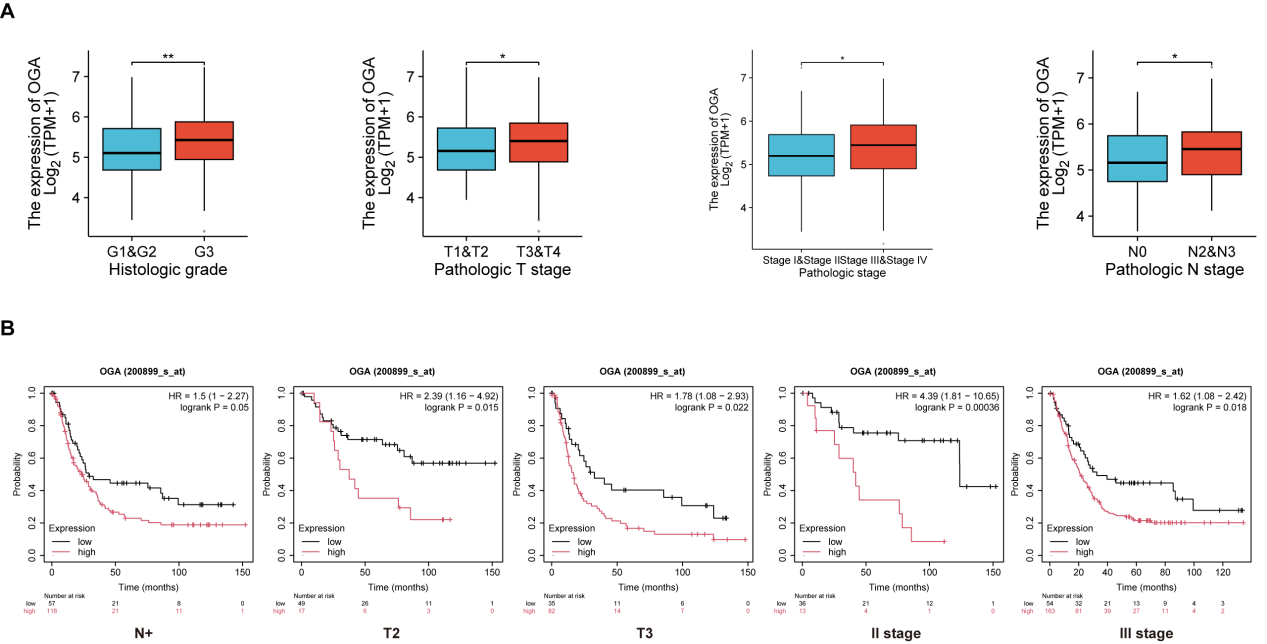


**Figure S7. High OGA Expression is Associated with Poor Prognosis in GC Patients. A)** Elevated OGA expression is associated with poor clinicopathological factors in patients with GC from the TCGA database. **B)** Kaplan–Meier curves of OS in subgroups of patients with GC based on the OGA expression levels. Data are expressed as mean ± SD of biological replicate experiments. *p < 0.05, **p < 0.01.
