# Supplementary figures and images for: O‐GlcNAcylation Regulation of SNAP29‐Dependent Autophagy Activation Dictates Chemoresistance in Gastric Cancer
Source: Adv Sci (Weinh). 2026 Jul 23:e76730. Online ahead of print. doi: 10.1002/advs.76730 (PMC13393265; doi:10.1002/advs.76730)

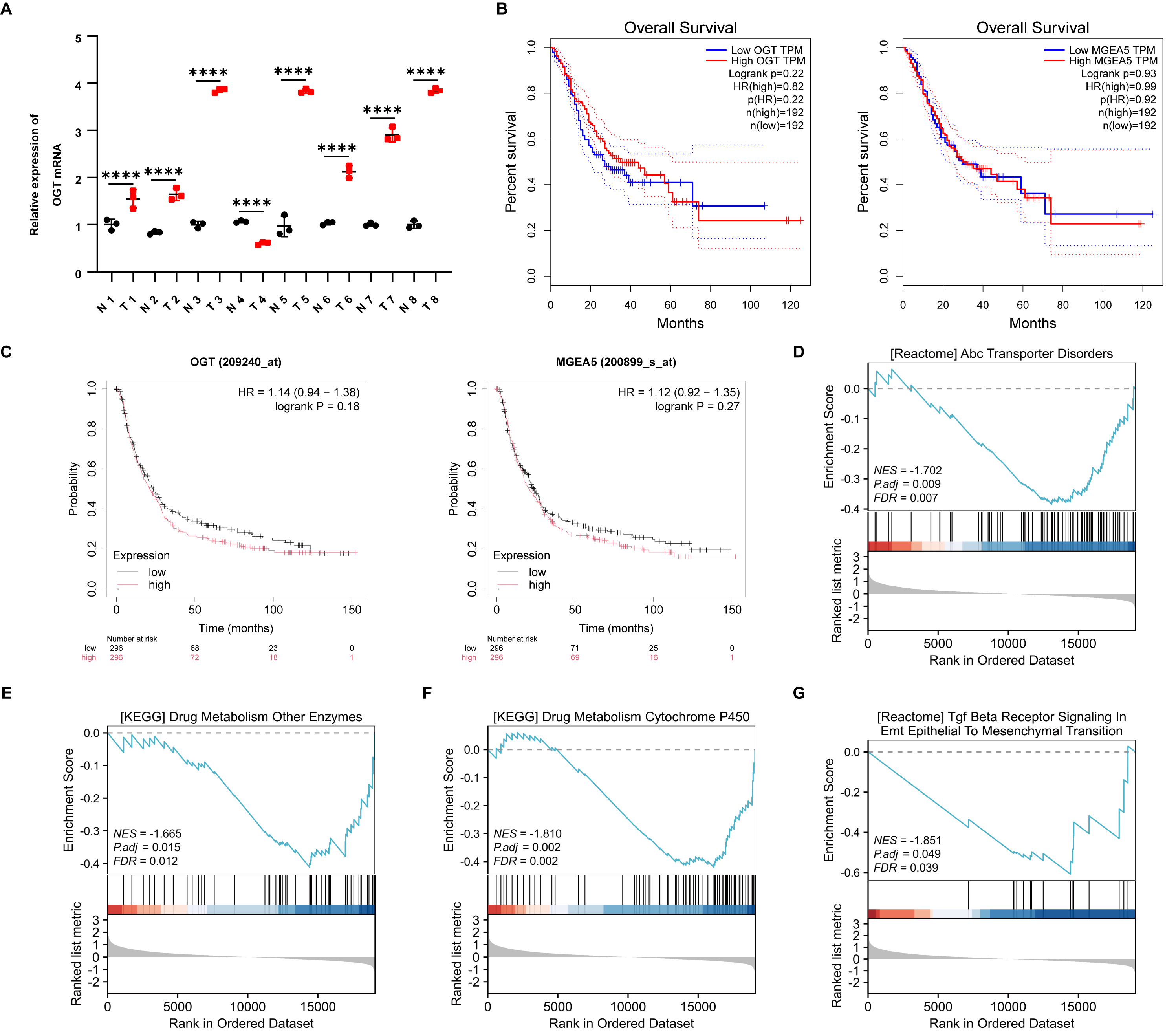

Supplement: Supplementary file 2 — Supporting File 2: advs76730‐sup‐0002‐FigureS1‐S7.zip. [file ADVS-9999-e76730-s001.zip › Supplementary Figure1.tif]

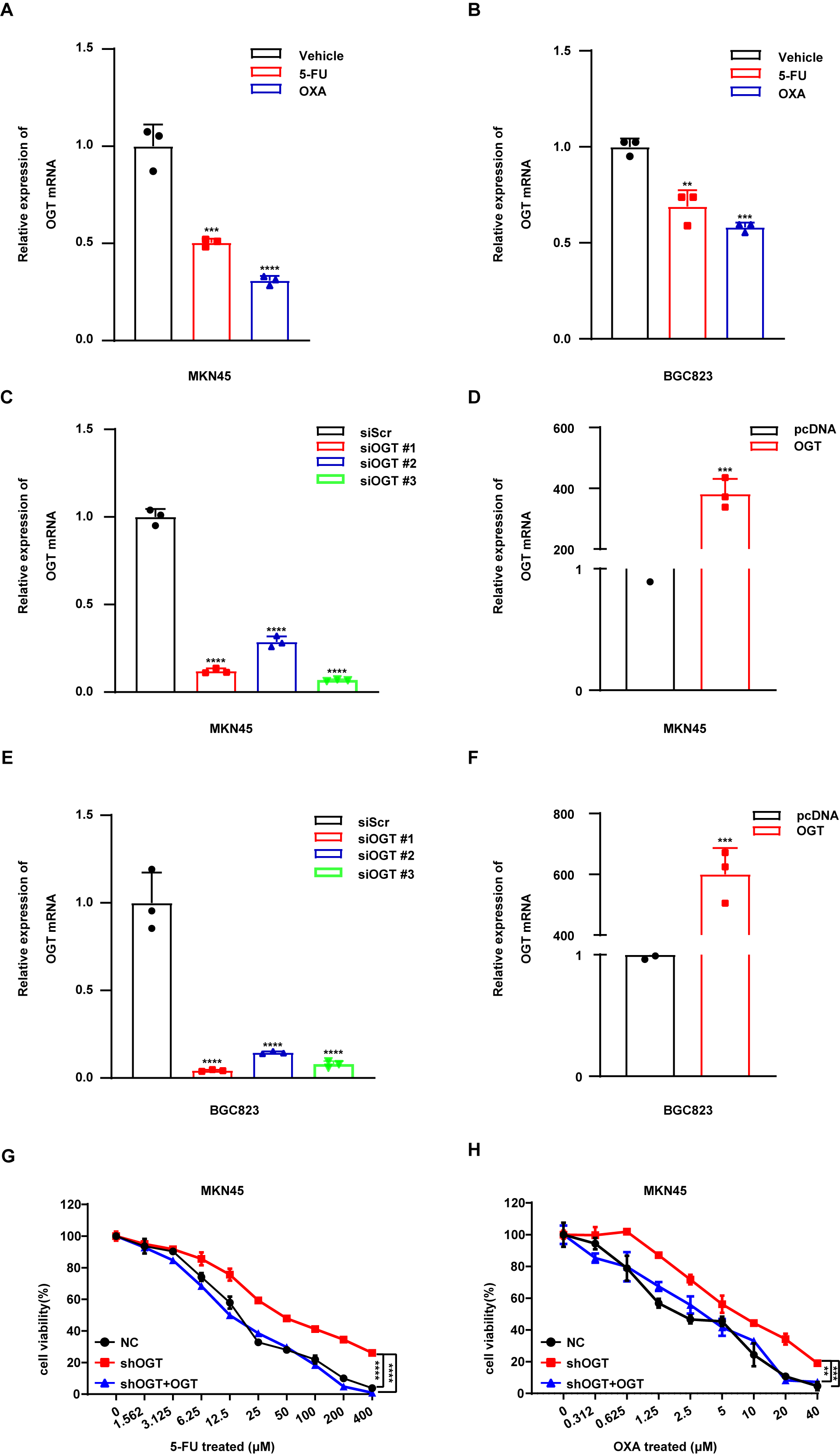

Supplement: Supplementary file 2 — Supporting File 2: advs76730‐sup‐0002‐FigureS1‐S7.zip. [file ADVS-9999-e76730-s001.zip › Supplementary Figure2.tif]

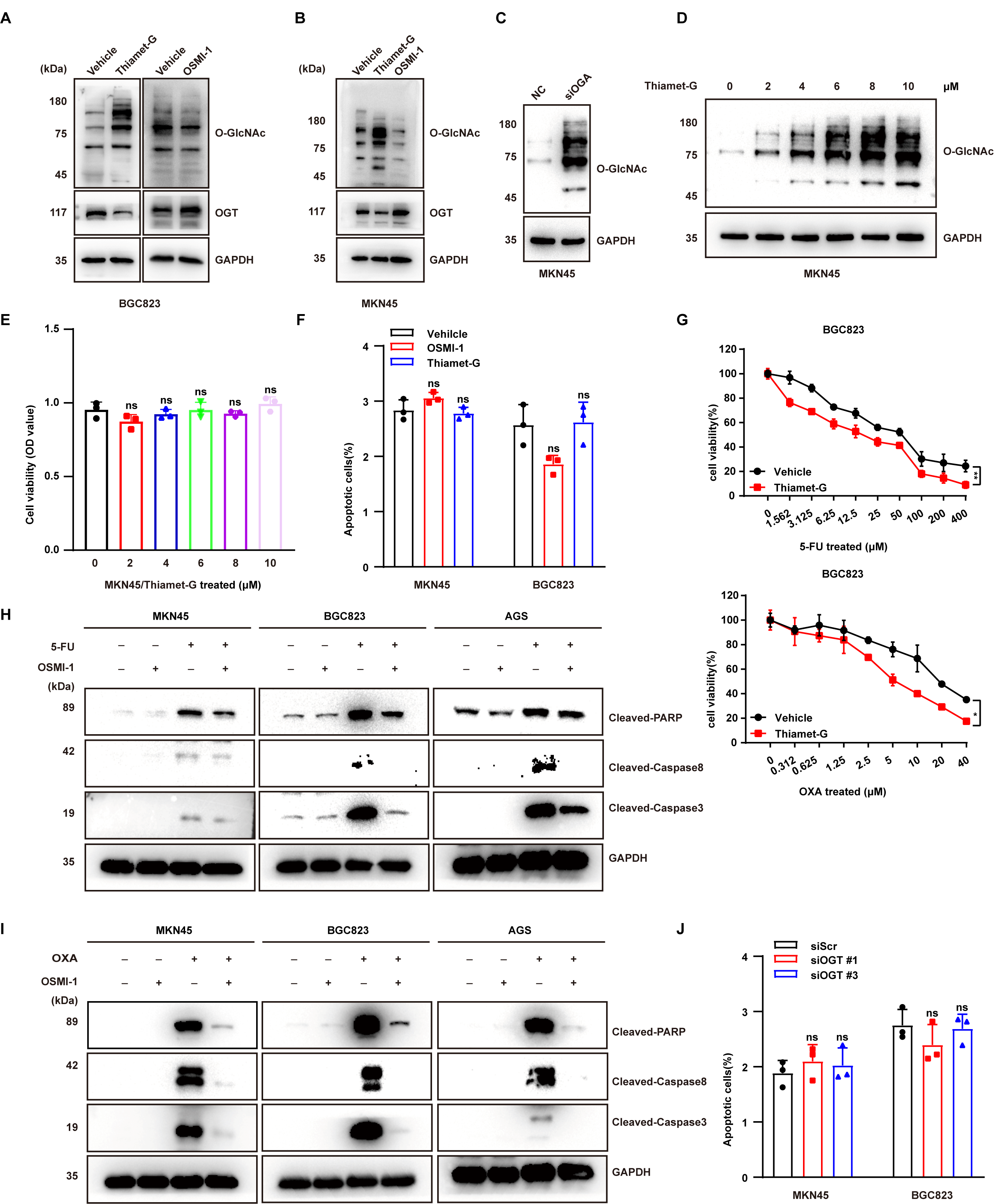

Supplement: Supplementary file 2 — Supporting File 2: advs76730‐sup‐0002‐FigureS1‐S7.zip. [file ADVS-9999-e76730-s001.zip › Supplementary Figure3.tif]

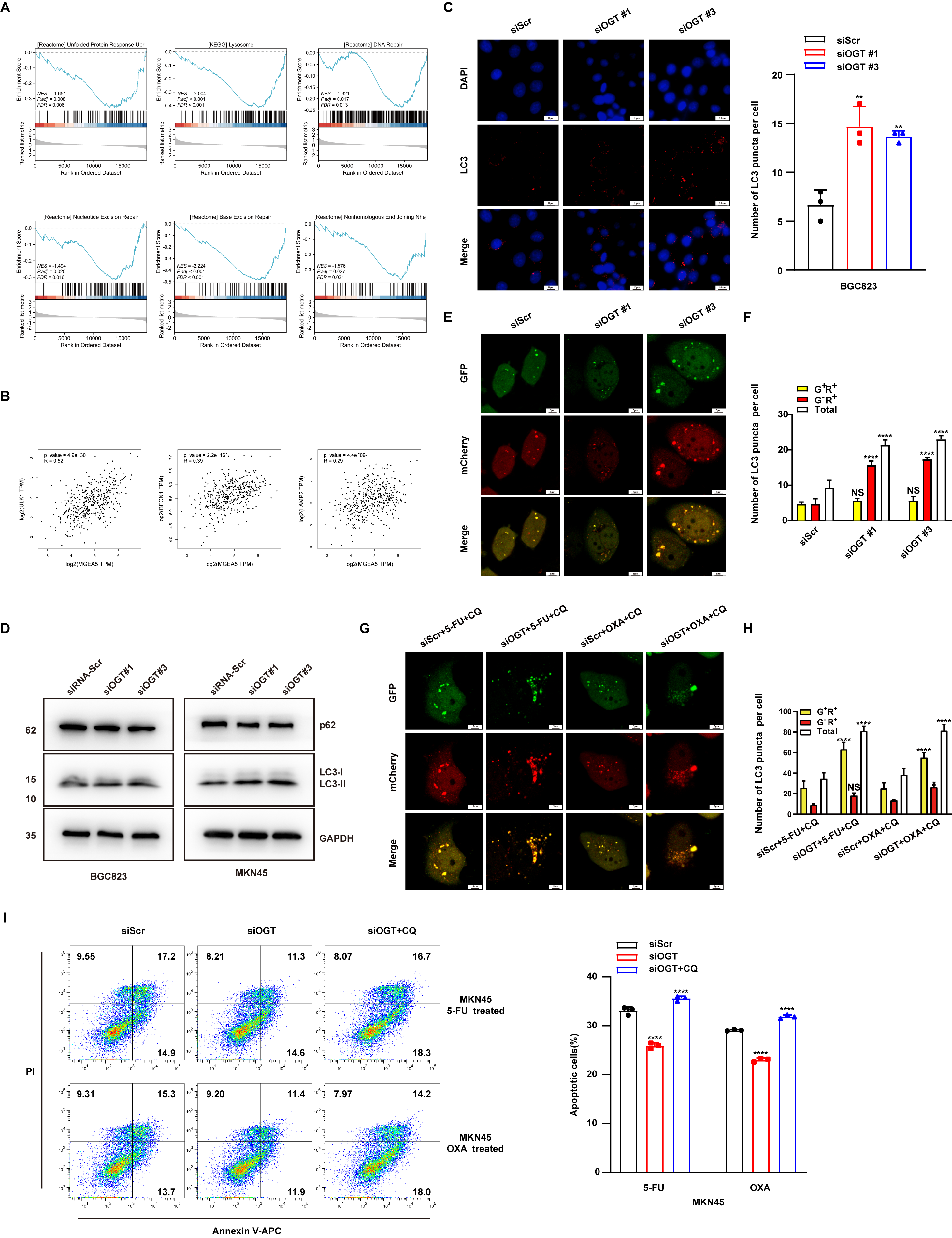

Supplement: Supplementary file 2 — Supporting File 2: advs76730‐sup‐0002‐FigureS1‐S7.zip. [file ADVS-9999-e76730-s001.zip › Supplementary Figure4.tif]

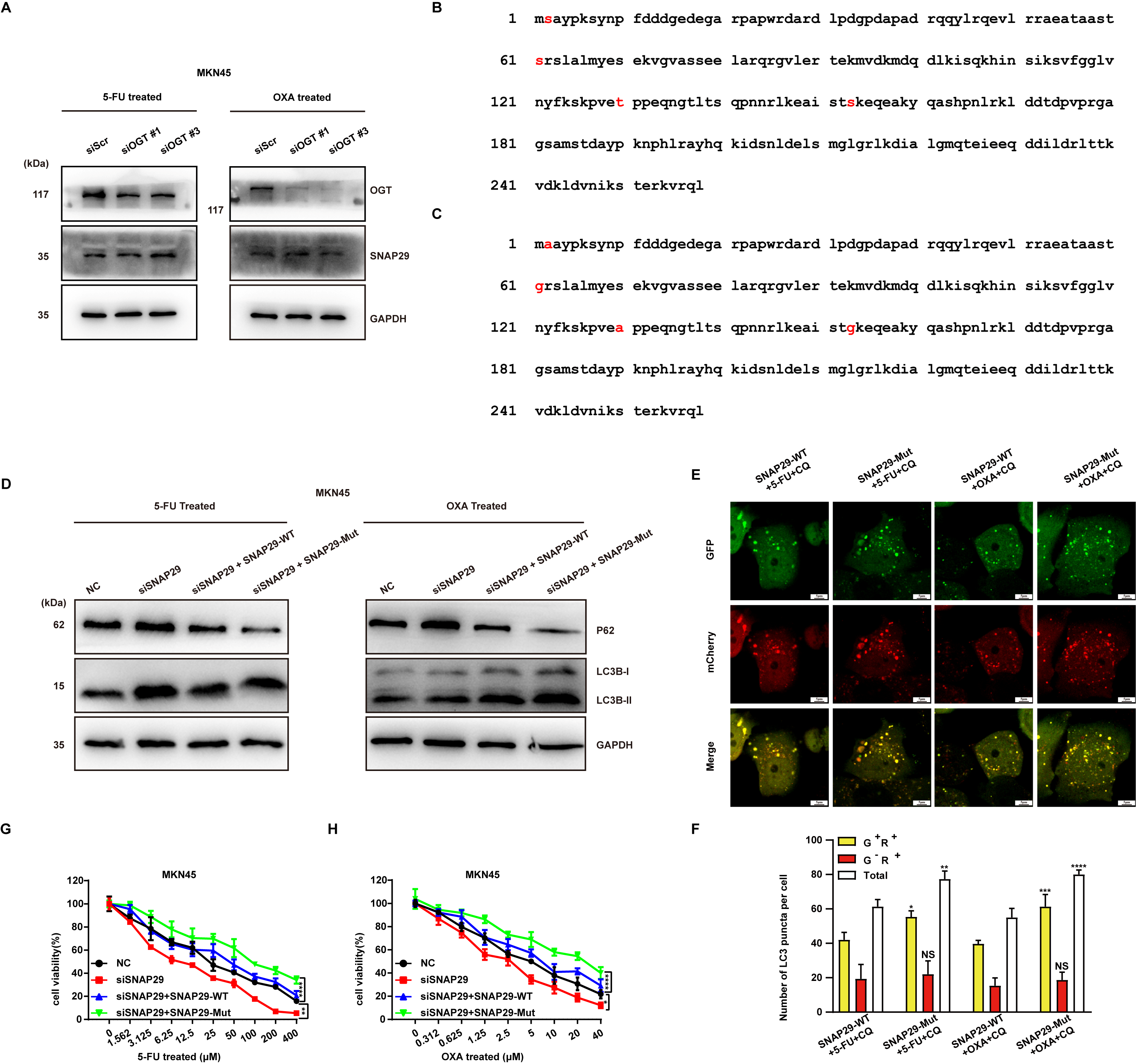

Supplement: Supplementary file 2 — Supporting File 2: advs76730‐sup‐0002‐FigureS1‐S7.zip. [file ADVS-9999-e76730-s001.zip › Supplementary Figure5.tif]

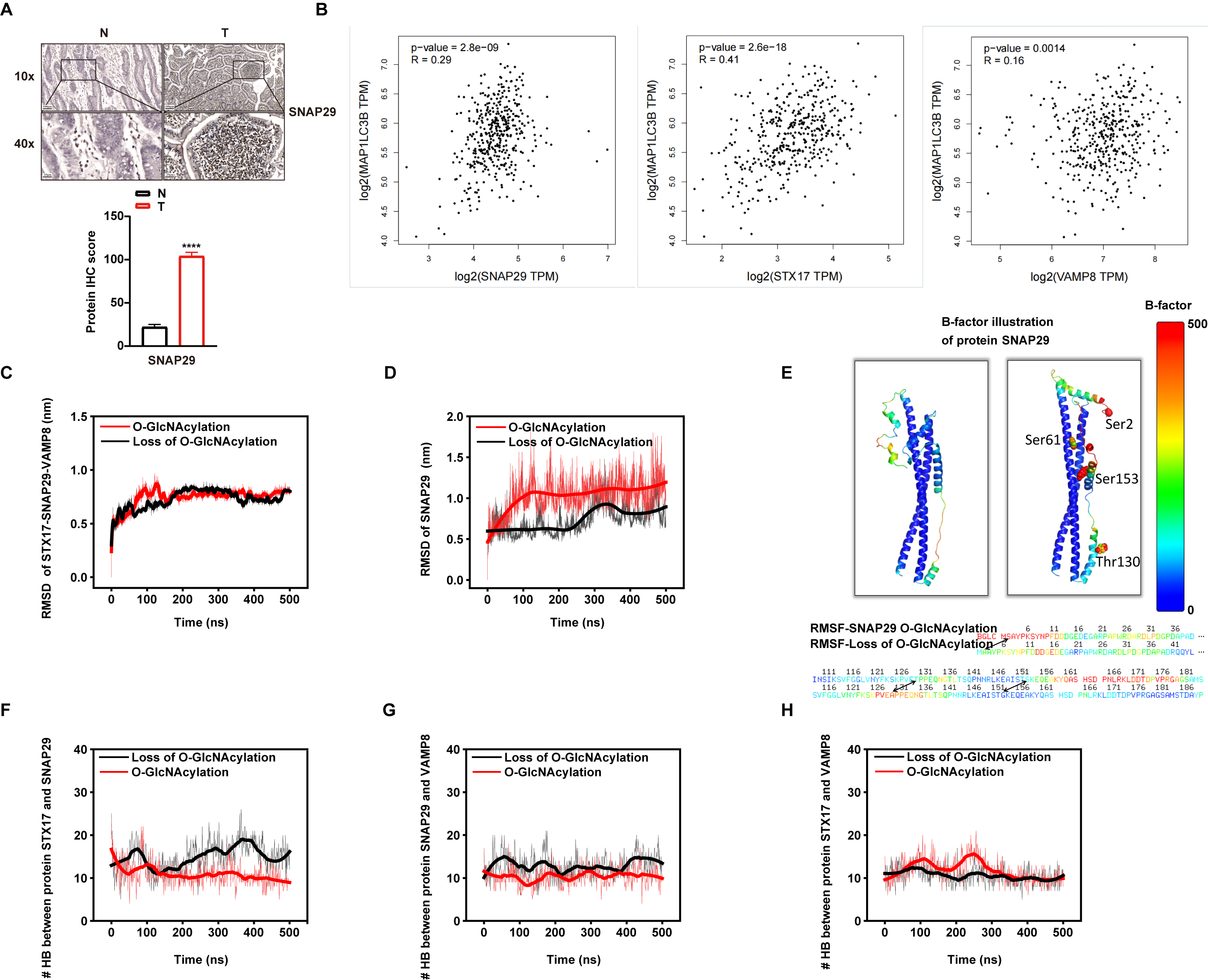

Supplement: Supplementary file 2 — Supporting File 2: advs76730‐sup‐0002‐FigureS1‐S7.zip. [file ADVS-9999-e76730-s001.zip › Supplementary Figure6.tif]

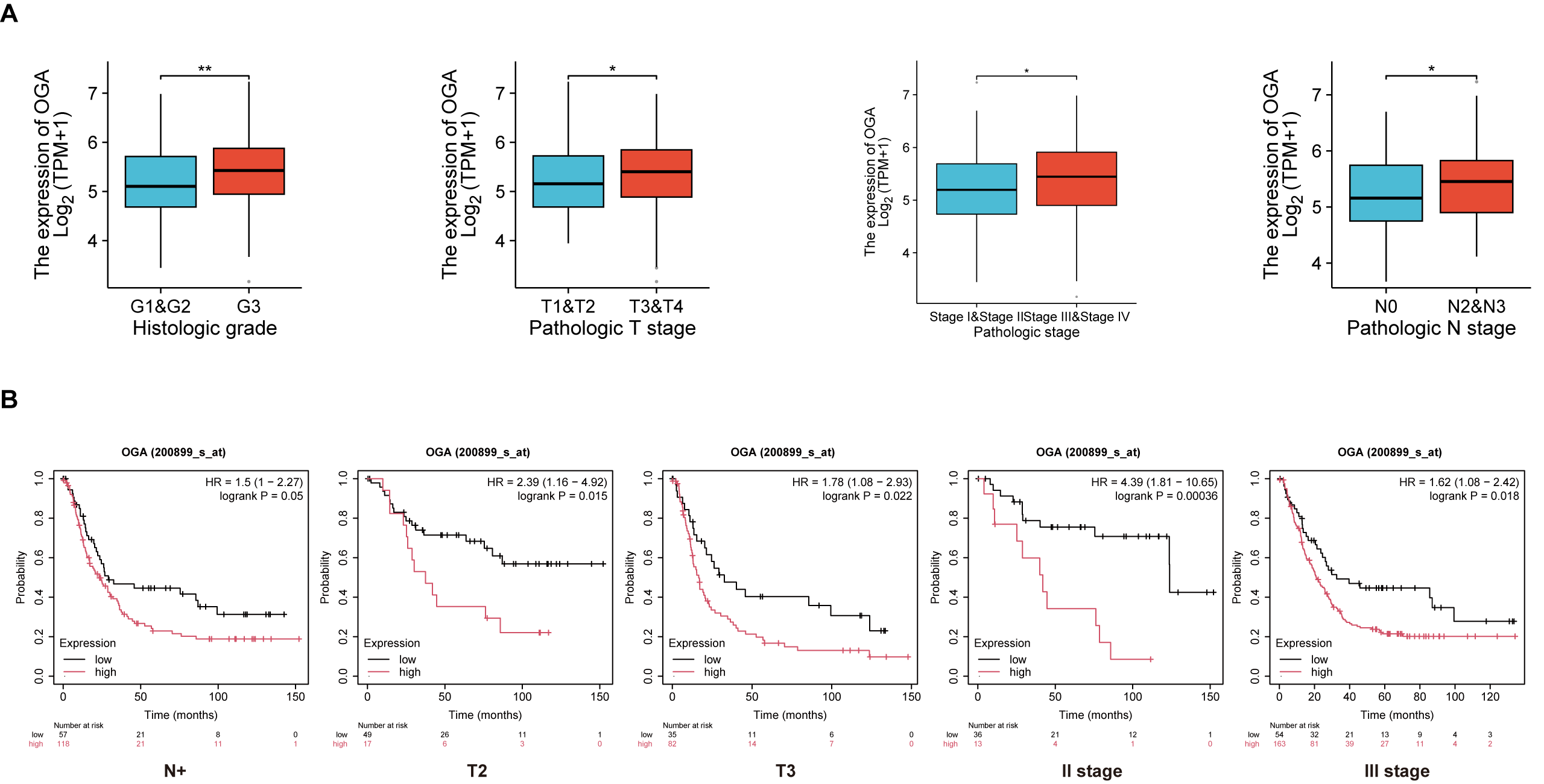

Supplement: Supplementary file 2 — Supporting File 2: advs76730‐sup‐0002‐FigureS1‐S7.zip. [file ADVS-9999-e76730-s001.zip › Supplementary Figure7.tif]
