# Supplementary material for: O‐GlcNAcylation Regulation of SNAP29‐Dependent Autophagy Activation Dictates Chemoresistance in Gastric Cancer
Source: Adv Sci (Weinh). 2026 Jul 23:e76730. Online ahead of print. doi: 10.1002/advs.76730 (PMC13393265; doi:10.1002/advs.76730)
Supplement: Supplementary file 3 — Supporting File 3: advs76730‐sup‐0003‐TableS1.docx. [file ADVS-9999-e76730-s003.docx]

**Supplementary Table 1.** Summary of Reagents and Materials

| **Reagents or Materials** | **Source** | **Cat. No.** |
| --- | --- | --- |
| **Antibodies** |  |  |
| Anti-OGT | Abcam | ab96718 |
| Anti-O-Linked N-Acetylglucosamine(RL2) | Abcam | ab2739 |
| Anti-SNAP29 | Abcam | ab138500 |
| Anti-SNAP29 | Abcam | Ab181151 |
| Anti-Syntaxin-17(STX17) | MBL | PM076 |
| Anti-VAMP8 | Abcam | ab76021 |
| Anti-LC3B | Abclonal | A19665 |
| Anti-LC3B | CST | 2775S |
| Anti-SQSTM1/p62 | Abclonal | A19700 |
| Anti-GAPDH | Proteintech | 60004-1-Ig |
| Anti-Mouse IgG, HRP-linked Antibody | CST | 7076s |
| Anti-Rabbit IgG, HRP-linked Antibody | CST | 7074s |
| IPKine™ HRP, Goat Anti-Mouse IgG LCS | Abbkine | A25012 |
| IPKine™ HRP, Goat Anti-Rabbit IgG LCS | Abbkine | A25222 |
| AffiniPure rabbit IgG (H+L) | BOSTER | BA1045 |
| Anti-Cleaved PARP | CST | 5625S |
| Anti-Cleaved Caspase-8 | CST | 9496 |
| Anti-Cleaved Caspase 3 | CST | 25128-1-AP |
| **Primers** |  |  |
| Human OGT forward primer 5'- AGTTCGTGGCAAAGTCTGGA -3' | TIANYI HUIYUAN | customized |
| Human OGT reverse primer 5'- ACCGTCATGGTCTTTGTAGTCC -3' | TIANYI HUIYUAN | Customized |
| Human SNAP29 forward primer 5'- CCAGTAGAGACCCCACCTGA -3' | TIANYI HUIYUAN | customized |
| Human SNAP29 reverse primer 5'- ATCTGTTGTTGGGCTGGGAG -3' | TIANYI HUIYUAN |  |
| Human SNAP29(Mut) forward primer 5'- ATCTGTTGTTGGGCTGGGAG -3' | TIANYI HUIYUAN | customized |
| Human SNAP29(Mut) reverse primer 5'- TGGGGACAGGGTCTGTATCA -3' | TIANYI HUIYUAN | customized |
| Human STX17 forward primer 5'- CCCGGCGGGAGGTTTTT -3' | TIANYI HUIYUAN | customized |
| Human STX17 reverse primer 5'- ATAGCTGGTTCAAGACGGCG -3' | TIANYI HUIYUAN | customized |
| Human VAMP8 forward primer 5'- AATGATCGTGTGCGGAACCT -3' | TIANYI HUIYUAN | customized |
| Human VAMP8 reverse primer 5'- GTGCTCAGATGTGGCTTCCA -3' | TIANYI HUIYUAN | customized |
| Human GAPDH forward primer 5'- GACAGTCAGCCGCATCTTCT -3' | TIANYI HUIYUAN | customized |
| Human GAPDH reverse primer 5'- GCGCCCAATACGACCAAATC -3' | TIANYI HUIYUAN | customized |
